# Men's nutrition knowledge is important for women's and children's nutrition in Ethiopia

**DOI:** 10.1111/mcn.13062

**Published:** 2020-08-04

**Authors:** Ramya Ambikapathi, Simone Passarelli, Isabel Madzorera, Chelsey R. Canavan, Ramadhani A. Noor, Semira Abdelmenan, Dagmawit Tewahido, Amare Worku Tadesse, Lindiwe Sibanda, Simbarashe Sibanda, Bertha Munthali, Tshilidzi Madzivhandila, Yemane Berhane, Wafaie Fawzi, Nilupa S. Gunaratna

**Affiliations:** ^1^ Department of Nutrition Science Purdue University West Lafayette Indiana USA; ^2^ Departments of Nutrition Harvard T.H. Chan School of Public Health Boston Massachusetts USA; ^3^ Departments of Global Health and Population Harvard T.H. Chan School of Public Health Boston Massachusetts USA; ^4^ Addis Continental Institute of Public Health Addis Ababa Ethiopia; ^5^ Food, Agriculture and Natural Resources Policy Analysis Network Pretoria South Africa; ^6^ Departments of Epidemiology Harvard T.H. Chan School of Public Health Boston Massachusetts USA; ^7^ Department of Public Health Purdue University West Lafayette Indiana USA

**Keywords:** dietary diversity, Ethiopia, men's nutrition knowledge, nutrition‐sensitive agriculture, women's nutrition knowledge

## Abstract

In an effort to address undernutrition among women and children in rural areas of low‐income countries, nutrition‐sensitive agriculture (NSA) and behaviour change communication (BCC) projects heavily focus on women as an entry point to effect nutritional outcomes. There is limited evidence on the role of men's contribution in improving household diets. In this Agriculture to Nutrition trial (Clinicaltrials.gov identifier: NCT03152227), we explored associations between men's and women's nutritional knowledge on households', children's and women's dietary diversity. At the midline evaluation conducted in July 2017, FAO's nutrition knowledge questionnaire was administered to male and female partners in 1396 households. There was a high degree of agreement (88%) on knowledge about exclusive breastfeeding between parents; however, only 56–66% of the households had agreement when comparing knowledge of dietary sources of vitamin A or iron. Factor analysis of knowledge dimensions resulted in identifying two domains, namely, ‘dietary’ and ‘vitamin’ knowledge. Dietary knowledge had a larger effect on women's and children's dietary diversities than vitamin knowledge. Men's dietary knowledge had strong positive associations with households' dietary diversity scores (0.24, *P* value = 0.001), children's dietary diversity (0.19, *P* value = 0.008) and women's dietary diversity (0.18, *P* value < 0.001). Distance to markets and men's education levels modified the effects of nutrition knowledge on dietary diversity. While previous NSA and BCC interventions predominantly focused on uptake among women, there is a large gap and strong potential for men’s engagement in improving household nutrition. Interventions that expand the role of men in NSA may synergistically improve household nutrition outcomes.

**Key messages**

- There is very little focus on men's role in women's and children's dietary outcomes in low‐income settings.
- Within households, men and women have high knowledge and agreement on optimal breastfeeding practices. However, there is low knowledge and agreement between men and women on complementary feeding, iron‐deficiency anaemia and vitamin A deficiency.
- Two components of nutrition knowledge (dietary and vitamin) among men and women were associated with higher dietary diversities of women, children and households.
- Men's nutrition knowledge had significant, positive and additive associations with households', children's and women's dietary diversity after adjusting for household wealth, women's education and nutrition knowledge.
- Targeted research exploring how nutrition knowledge is gendered and how to engage men in nutrition programming may lead to better outcomes.

## 1 INTRODUCTION

Nutrition interventions, including a large number of nutrition‐sensitive agriculture (NSA) programmes, focus on women as an entry point to effect positive nutritional outcomes. By default, men have been mostly left out from the design and implementation of NSA programmes because nutrition is typically perceived to bea woman's domain. In particular, NSA programmes often focus on improving women's nutrition knowledge and empowerment to improve their decision‐making power for food purchases and allocation of nutritious food (Ruel, Alderman, Maternal, & Child Nutrition Study, 2013; Ruel, Quisumbing, & Balagamwala, 2018).

**FIGURE 1:**
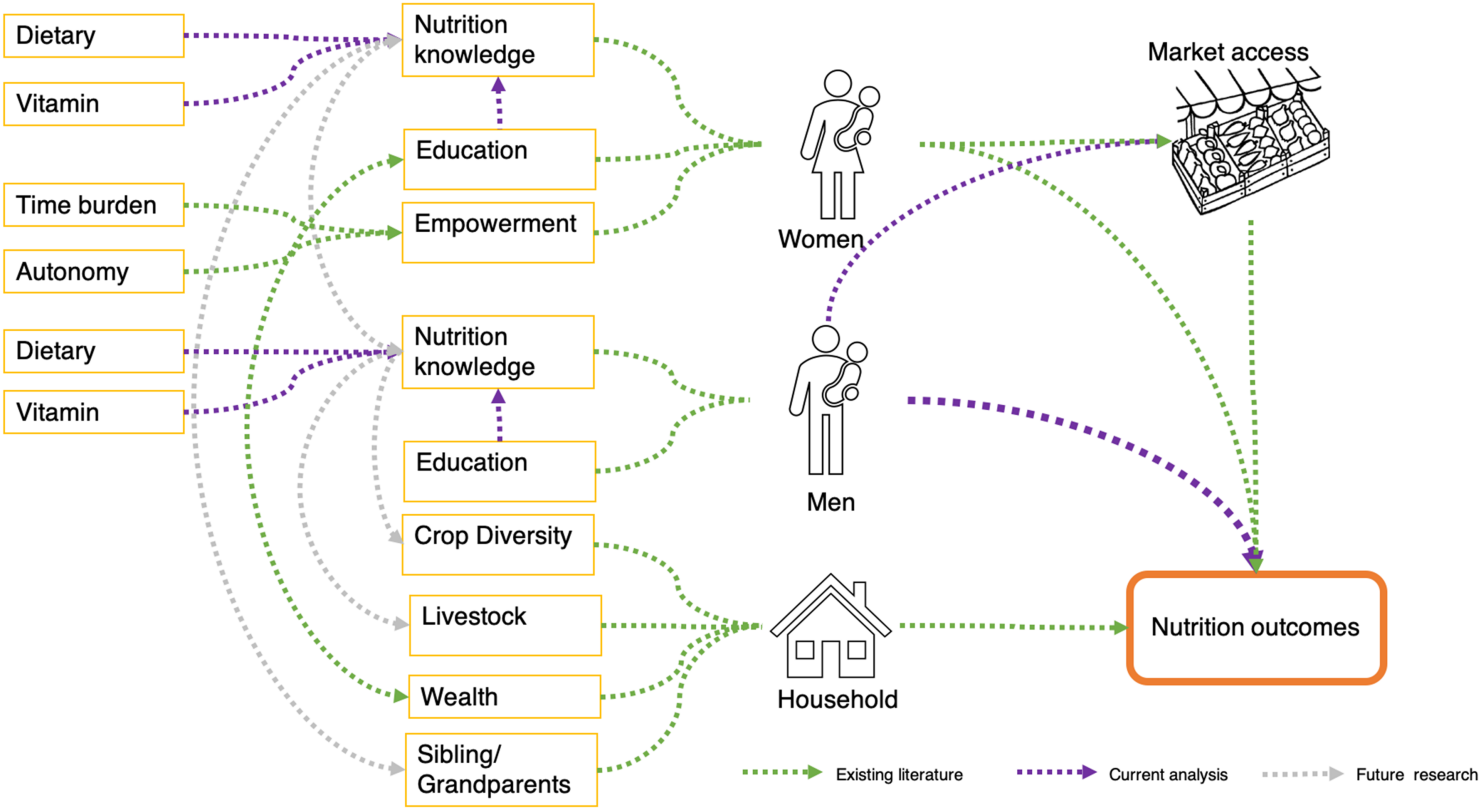
Hypothesized pathways from nutrition knowledge to nutrition outcomes

Women's empowerment, through autonomy over household purchases, is positively associated with children's nutritional status in Ethiopia (Abate & Belachew, 2017) and is associated with women's dietary diversity in Ghana (Amugsi, Lartey, Kimani, & Mberu, 2016). One study found that engaging husbands during pregnancy resulted in higher dietary diversity among women in Bangladesh, but it is unknown whether these effects are sustained after pregnancy or observed among non‐pregnant or lactating women (Nguyen et al., 2018). Women's empowerment, however, cannot be achieved without equitable contribution from men, especially in their roles as fathers, husbands, household heads and, more importantly, prominent players in decision‐making on income, food purchases, and consumption (Engle, 1997). Despite the central role of men, very few studies have evaluated the impact of men's engagement on household nutrition, including diets and nutritional status of women in low resource settings (Schneider & Masters, 2018). Highlighted in Figure 1 are the hypothesized pathways from nutrition knowledge to household nutrition outcomes based on existing literature (green), current analysis (purple) and proposed future research (grey). We have aligned some of these pathways with theongoing and innovative work on Women's Empowerment in Nutrition dimensions, with a focus on knowledge, agency, and resources (Narayanan, Lentz, Fontana, De, & Kulkarni, 2019).

Women's nutrition knowledge is strongly associated with children's dietary diversity, nutritional status and micronutrient intake (Block, 2004, 2007; Cunningham et al., 2017; Debela, Demmler, Rischke, & Qaim, 2017; Fadare, Amare, Mavrotas, Akerele, & Ogunniyi, 2019; Monteban, 2017; Oduor, Boedecker, Kennedy, Mituki‐Mungiria, & Termote, 2018; Ruel, Habicht, Pinstrup‐Andersen, & Grohn, 1992). However, the association between women's knowledge and her own diet and nutritional status remains unknown (Cunningham et al., 2017; Fadare et al., 2019; Schneider & Masters, 2018; Williams, Campbell, Abbott, Crawford, & Ball, 2012). An innovative study in Northern Ethiopia found that fathers' nutrition knowledge and education was associated with higher dietary diversity among children but did not account for mothers' knowledge or education (Bilal et al., 2016). Taken together, these studies suggest that nutrition knowledge (mostly women's) is necessary but not sufficient for optimal nutrition outcomes (mostly children's) and that there may be other contributing factors such as education (women's and men's), household wealth and access to markets to leverage higher gains from knowledge to nutrition outcomes (Burchi, 2010; Hirvonen, Hoddinott, Minten, & Stifel, 2017; Ruel et al., 1992). Additionally, the importance of the nutrition knowledge of other family members, such as grandparents, for child outcomes has been explored extensively (Karmacharya, Cunningham, Choufani, & Kadiyala, 2017). Informational flow between grandparents and mothers occurs; however, nutrition knowledge flow between mothers and older children (siblings) on their own nutrition or younger children's nutrition outcomes remains to be explored.

Distillation of these studies conducted in low‐income settings points to three substantial gaps. First, the impact of men's (fathers'/spouses') nutrition knowledge on women's and children's nutrition outcomes remains under‐studied. Second, an understanding of how men's and women's nutrition knowledge within a household are associated for optimal nutrition outcomes remains unknown. Lastly, components of nutrition knowledge associated with the highest gains in nutrition outcomes need to be identified.

To address these research gaps, we used data from Agriculture to Nutrition (ATONU) study (Clinicaltrials.gov identifier: NCT03152227) ‐‐ a cluster randomized trial conducted in Ethiopia. The main objectives of this paper are: (1) to describe men's and women's nutrition knowledge and agreement between these two within a household; (2) to examine how nutrition knowledge of both men and women is associated with households', children's and women's dietary diversity after adjusting for men's and women's education, household wealth and size, and village‐level clustering; and (3) to identify components of nutrition knowledge with the highest effect size on nutrition outcomes.

## 2 METHODS

### 2.1 Study setting

We used data obtained from ATONU, a cluster randomized trial that was nested within the African Chicken Genetic Gains (ACGG) project and has been described previously (Ambikapathi et al., 2019; Dessie, 2016). The trial began in 2016 with 21 months of intervention activities across four regions of Ethiopia, including Tigray, Amhara, Oromia and Southern Nations, Nationalities, Peoples' Region (SNNPR). Interventions included the introduction of 25 chickens of improved breeds per household (arm 1, ‘ACGG’); behaviour change communication on women and children's nutrition, water, sanitation, hygiene, and women's empowerment, plus the 25 improved chickens (arm 2, ‘ACGG + ATONU’); and lastly, a no intervention arm (arm 3, ‘control’). Villages, the primary sampling units, were randomly selected, and stratified by district and agro‐ecological zone.

At the baseline evaluation, 2,117 households were enrolled in the study. Households meeting the following inclusion criteria were eligible to be enrolled in the study: (1) have a woman of reproductive age (18–45 years), (2) provide informed consent, and (3) participated in chicken farming for the last 2 years and currently have less than 50 chicken (same criteria for the ACGG programme). Surveys were administered to the household head and one woman of reproductive age. Among households with children under 36 months, one eligible child was picked at random for anthropometry, morbidity and dietary diversity assessments.

The current analysis uses data from the midline evaluation because nutrition knowledge surveys were only added at this evaluation. The survey was conducted from July to August 2017 on 2,042 households (75 were lost to follow‐up from baseline). For the purposes of this analysis, only households with a married couple (e.g., male household heads married to women) who answered the nutrition knowledge surveys were included; hence, 646 households were excluded for the following reasons: 274 woman‐headed households, 347 respondents in a non‐marital relationship with the household head and 25 surveys with missing data. The excluded 274 women‐headed households did not vary significantly with regard to the three main outcomes (women's, children's and household dietary diversity scores). In total, 1,396 households with 743 children were included in the analysis.

Physical access to market in terms of duration (minutes to travel from the household to the market) was available only among 84% of the sample population and was limited to three regions (Amhara, Oromia and SNNPR) at the midline evaluation; therefore, market access was included in a subset analysis. Food security was measured using the Household Food Insecurity Access Scale (HFIAS; Coates, Swindale, & Bilinsky, 2007). WHO/UNICEF definitions (2015) were used to estimate the prevalence of improved access to water and sanitation. Household wealth quintiles were developed based on assets, land ownership, and household characteristics (Ambikapathi et al., 2019).

### 2.2 Key exposures: Nutrition knowledge definitions

Nutrition knowledge of the study participants was assessed using the Food and Agriculture Organization's (FAO) nutrition‐related knowledge, attitudes and practices questionnaire (Marías & Glasauer, 2014). Out of 13 available modules, we used five modules on breastfeeding, infant feeding, nutrition during pregnancy and lactation, iron deficiency and vitamin A deficiency for analysis.. These questions have multiple correct answers listed. Responses were recorded by the survey team if the respondent gave one of the listed answers; responses not listed were entered as text in the ‘other’ category and were analysed for correctness. Responses within knowledge questions were summarized. For example, there are six correct answers for ‘ways to provide good nutrition for pregnant/lactating women’ (eating more food, eating more at each meal, eating more frequently, eating more protein‐rich foods, eating iron‐rich foods and using iodized salt for preparing meals; Marías & Glasauer, 2014). Each item was given 1 point, yielding a maximum possible score of 6. In total, there were four nutrition knowledge variables per woman and man: (1) ways to provide good nutrition for pregnant/lactating women, (2) ways to improve diets for children, (3) knowledge of vitamin A‐rich foods and (4) knowledge of iron‐rich foods.

**FIGURE 2:**
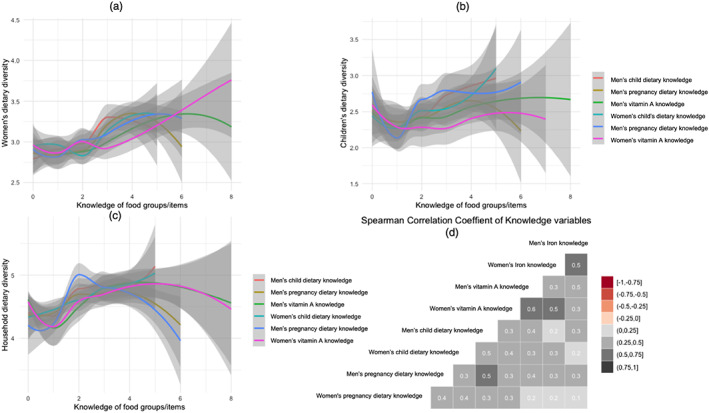
Panels examining the relationship between men's and women's nutrition knowledge and (a) women's, (b) children's, and (c) households' dietary diversity, and (d) Spearman's correlation matrix of nutrition knowledge variables. Grey shading in (a)–(c) indicates standard error of the loess curves. Grey region on each of the loess curves indicates the standard error

Because these four knowledge variables were highly correlated with each other, exploratory factor analysis was utilized to distil nutrition knowledge variables (Figure 2d). Previous research assessing mothers' knowledge of child nutrition have used similar data reduction approaches (Fadare et al., 2019; Hirvonen et al., 2017). Based on iterative factor analyses (run separately for women and men), two factor models were used, and they explained approximately 75% of the variance in the distilled nutrition knowledge variables. Factor loadings and scores are presented in Table S1. Exploratory factor analysis on nutrition knowledge variables uniquely loaded on two sets of factor groups (factor loadings > 0.3) that were similar for both men and women. This included (1) a ‘dietary knowledge’ factor, which had high factor loadings on procedural knowledge to improve nutrition for women and children and (2) a ‘vitamin knowledge’ factor, which had high factor loadings on food groups that are rich with vitamin A or iron (Velardo, 2015). Standardized regression scores for men and women were used as the main nutrition knowledge exposures.

### 2.3 Key outcome variables

There were three main outcome variables: household dietary diversity scores among households (HDDS, 1‐month recall, 10 food groups), children's dietary diversity (CDDS, 1‐day recall, seven food groups) and women's dietary diversity (MDD‐W, 1‐day recall, 10 food groups; FAO & FHI 360, 2016; World Health Organization, 2010; Swindale & Bilinsky, 2006). Less than 5% of the sampled women mentioned that day of dietary data collection was a holiday, whereas 24% mentioned they fasted (did not consume animal source foods according to the Ethiopian Orthodox tradition). There were no significant differences in MDD‐W by fasting, likely because of very low intakes of animal source foods. We made a change to HDDS by extending the recall from one day to one month to examine typical food access and because there was low food diversity in these settings. Finally, to examine the specificity of knowledge of food groups to a behaviour, we evaluated the impact of knowledge factors on consumption of individual food groups for women.

### 2.4 Statistical analysis

For comparison of intervention arms, joint *F* tests were obtained from generalized linear mixed models adjusting for clustering at the village level. Linear polynomial regression was used to visualize the relationships between dietary diversity scores and knowledge variables (Figure 2). Spearman rank correlation was used to examine correlations within the eight nutrition knowledge variables. Mixed effects linear and logistic regression models adjusting for village‐ (*kebele*, lowest administration unit in Ethiopia) level clustering were used to evaluate the associations between exposures and continuous and binary outcomes. All models were adjusted for household size, wealth quintiles, woman's age and education, man's age and education and the four geographical regions. Models with CDDS were adjusted for child age. Education is often associated with nutrition literacy and uptake^1992^, and therefore analysis examining the interaction between education and nutrition knowledge was explored in the multivariable models 1992. Treatment arms were not significant in all models, therefore removed from the main models. Summary data are presented below as median with first and third quartiles (interquartile range [IQR]: Q1, Q3) or as percentages.

### 2.5 Ethical considerations

The study protocol was approved by the Institutional Review Board of the Harvard T.H. Chan School of Public Health and the Ethical Committee at Addis Continental Institute of Public Health. All participants provided written informed consent; if the participant was unable to sign, a thumb print signature was obtained from the participant.

## 3 RESULTS

The median age of women included in this analysis was 34 years (IQR: 28, 39), and over half (60%) of women had no schooling, whereas the median age of men was 40 years (IQR: 35, 48), and a quarter (27%) of the men had no schooling (Table 1). Median age of the children was 22 months. Women in the control arm were on average younger, by 3 years, than women in the intervention arms. Seventy‐nine percent of the households had access to improved water, whereas only one third of households had access to improved sanitation. The median time to the closest market was 45 min (IQR: 30, 60) and about half of the households reported that they attend the markets weekly. Half (52%) of the households reported having food access security.

**TABLE 1:** Demographics and main variables of interest from the ATONU study midline evaluation, July to August 2017, Ethiopia

		ACGG	ACGG + ATONU	Control	Total	*P* value
Level	Main outcomes and exposures	*N* = 434	*N* = 426	*N* = 536	*N* = 1,396	
	Child outcomes and exposures	*N* = 228	*N* = 208	*N* = 307	*N* = 743	
Household	Household dietary diversity score—1‐month recall^a^	4 (3, 6)	5 (3, 6)	4 (3, 6)	4 (3, 6)	0.52
Women	Women's dietary diversity score—24‐h recall	3 (2, 4)	3 (2, 4)	3 (2, 4)	3 (2, 4)	0.67
Women	% Consumption of meat (*n*)	2.3 (10)	3.8 (16)	2.6 (14)	2.9 (40)	0.60
Women	% Consumption of legumes (*n*)	56.2 (244)	53.5 (228)	49.8 (267)	52.9 (739)	0.88
Women	% Consumption of nuts (*n*)	4.1 (18)	2.1 (9)	2.6 (14)	2.9 (41)	0.60
Women	% Consumption of vitamin A‐rich foods (*n*)	5.3 (23)	4.7 (20)	5.4 (29)	5.2 (72)	0.97
Women	% Consumption of green leafy vegetables (*n*)	30.6 (133)	35.9 (153)	33.8 (181)	33.5 (467)	0.83
Women	% Consumption of eggs (*n*)	7.1 (31)	8.7 (37)	4.1 (22)	6.4 (90)	0.54
Women	% Consumption of dairy (*n*)	20.3 (88)	20.0 (85)	17.2 (92)	19.0 (265)	0.92
Women	% Women meeting minimum dietary diversity (binary, <5 food groups)	9.2 (40)	8.9 (38)	10.1 (54)	9.5 (132)	0.75
Child	Children's dietary diversity score with seven food groups (original indicator)	3 (2, 4)	3 (1, 4)	3 (2, 3)	3 (1, 3)	0.63
Child	% Children meeting minimum dietary diversity (<4 food groups)	27.6 (63)	25.4 (53)	20.5 (63)	24.1(179)	0.37
Child	% Consumption of meat (*n*)	1.8 (4)	1.0 (2)	1.6 (5)	1.5 (11)	0.77
Child	% Consumption of legumes (*n*)	42.5 (97)	32.2 (67)	34.8 (107)	36.4 (271)	0.64
Child	% Consumption of vitamin A‐rich foods (*n*)	25.4 (58)	28.3 (59)	26.3 (81)	26.6 (198)	0.70
Child	% Consumption of other fruits and vegetables (*n*)	58.3(133)	49.0 (102)	53.7 (165)	53.8 (400)	0.50
Child	% Consumption of eggs (*n*)	14.4 (33)	11.5(24)	6.8 (21)	10.5 (78)	0.13
Women	Women's age (years)	35 (29, 40)	35 (28, 40)	32 (27, 38)	34 (28, 39)	0.003
Men	Men's age (years)	42 (35, 48)	42 (35, 50)	40 (35, 48)	40 (35, 48)	0.18
Child	Children's age (months)	23 (15, 33)	23 (13, 33)	21 (13, 31)	22 (13, 32)	0.66
Women	Women's education^b^					
	No schooling	58.1 (252)	60.8 (259)	59.9 (321)	59.6 (832)	0.81
	Primary 1	20.5 (89)	18.0 (77)	19.2 (103)	19.3 (269)	
	Primary 2	12.4 (54)	12.0 (51)	15.7 (84)	13.5 (189)	
	Secondary 1, Secondary 2 and university	5.1 (22)	5.4 (23)	3.2 (17)	4.4 (62)	
	Religious school/literacy programme	3.9 (17)	3.8 (16)	2.0 (11)	3.2 (44)	
Men	Men's education^b^					
	No schooling	22.1 (96)	26.1 (111)	30.4 (163)	26.5 (370)	0.12
	Primary 1	23.0 (100)	24.6 (105)	25.9 (139)	24.6 (344)	
	Primary 2	31.3 (136)	29.6 (126)	25.8 (138)	28.7 (400)	
	Secondary 1, Secondary 2 and university	14.8 (64)	11.5 (49)	10.1 (54)	12.0 (167)	
	Religious school/literacy programme	8.8 (38)	8.2 (35)	7.8 (42)	8.2 (115)	
Household	% Access to improved water (*n*)	83.8 (364)	81.9 (349)	73.1 (392)	79.2 (1,105)	0.33
Household	% Access to improved sanitation (*n*)	32.7 (142)	30.8 (131)	35.8 (192)	33.3 (465)	0.95
Household	Size of land owned (*timad*; 4 *timads* = 1 hectare)	4 (2, 7)	4 (2, 6)	3 (2, 5)	4 (2, 6)	0.63
Household	Distance to the closest market (minutes, *n* = 1,171)	45 (30, 60)	40 (25, 60)	60 (30, 90)	45 (30, 60)	0.13
Household	Total number of HH members	7 (5, 8)	7 (5, 8)	6 (5, 8)	7 (5, 8)	0.10
Household	Food Insecurity Access (FIA) (%)					
	Food secure	54.8 (238)	52.1 (222)	48.3 (259)	51.6(719)	0.43
	Mildly food insecure	8.5 (37)	12.2 (52)	7.5 (40)	9.2 (129)	
	Moderate food insecure	19.6 (85)	18.3 (78)	23.3 (125)	20.6 (288)	
	Severe FIA	17.1 (74)	17.4 (74)	20.9 (112)	18.6 (260)	

Abbreviations: ACGG, African Chicken Genetic Gains; ATONU, Agriculture to Nutrition.

^a^ Summary data are either presented as median with quartiles 1 and 3 (Q1, Q3) or percentages within treatment arms with sample size in parentheses.

^b^ "Primary 1" refers to 1–5 years of schooling; "Primary 2" refers to 6–9 years of schooling; "Secondary 1" and "Secondary 2" refer to 10–17 years of schooling.

Median household dietary diversity scores were four food groups in ACGG and control arms, while the ACGG + ATONU arm had five food groups. The top five food groups consumed by the households in the last 30 days were grains (94%), legumes (69%), oils and fats (57%), dairy (42%) and eggs (40%). Less than 10% of women met the recommended dietary diversity (at least five food groups out of 10). Consumption of individual food groups for women are summarized in Table 1. Besides staples, women most commonly consumed legumes and green leafy vegetables, while very few women reported consuming meat, nuts or other vitamin A‐rich produce (mostly vitamin A‐rich vegetables) in the previous 24 h. Besides staples, children consumed foods from the fruits and vegetables food groups, followed by vitamin A‐rich foods, and other fruits and vegetables. Both women and children rarely consumed meat. Less than 7% of women and 11% of children had consumed eggs in the previous 24 h. Neither dietary diversity nor the consumption of individual foods was significantly different across treatment arms at midline evaluation for women and children.

There were regional differences in diets among women, children and households (see Table S2). Median HDDS and CDDS were five and three food groups in Amhara and Oromia. While in SNNPR and Tigray HDDS and CDDS were lower by one food group for HDDS and CDDS.. We saw similar trends in MDD‐W with SNNPR having one less food group compared to Tigray, Amhara and Oromia regions. Regional variations in consumption of food groups were also observed, for example, 70.2% (52.4% in children) of women in Amhara consumed pulses in the previous day compared with 32.0% (20.1% in children) in SNNPR. Median duration to the closest market was lowest in SNNPR at 30 min and highest in Amhara at 60 min.

The relationships between men's and women's nutrition knowledge and women's, children's and households' dietary diversity scores are shown in Figure 2. Nutrition knowledge of iron‐rich foods was not plotted because over 75% of the sampled participants (both men and women) could only list one correct answer. The grey shading around each of the loess curves indicates standard error (SE). Because very few participants had illustrated knowledge of four food groups, the SEs after four food groups are fairly large. Figure 2a shows the positive and mostly linear relationship between six nutrition knowledge variables and women's dietary diversity. Figure 2b shows the effect of men's child dietary knowledge (red line) on children's dietary diversity is higher compared with women's child dietary knowledge (blue line). In Figure 2c, the relationships between household dietary diversity and knowledge are shown. There is a curvilinear relationship with knowledge variables and household dietary diversity scores. Finally, Figure 2d provides Spearman's correlation matrix of the eight knowledge variables, highlighting two important structures in the knowledge data. First, there is a strong positive correlation between men and women for each type of knowledge. For example, men's vitamin A knowledge is highly correlated with women's vitamin A knowledge within the same household. Second, men's knowledge variables tend to be more correlated with each other than are women's knowledge variables.

Table 2 summarizes the nutrition knowledge responses between men and women within a household. Agreement within a household illustrates the knowledge gaps among men and women from the same households. In general, over 80–90% of men and women have high knowledge on exclusive breastfeeding and optimal breastfeeding practices. However, knowledge on food groups and dietary practices to improve nutrition among children and women is very low. There is also higher discordance of knowledge within households on nutrition practices related to women and children and on knowledge of foods rich in specific nutrients. For example, more than 45% of men and women have heard of vitamin A deficiency, but in only 27% of households both individuals have heard of vitamin A deficiency.

**TABLE 2:** Summary of nutrition knowledge questions and correct answers from women and men, agreement within household, and factor analysis grouping

Nutrition knowledge questions; *N* = 1,396^a^	Women's (%)	Men's (%)	Households with agreement on the correct answer (%)	Factor analysis grouping
What is the first food a newborn baby should receive? (correct answer: only breast milk/colostrum)	98.4	96.6	95.6	Not included
% of participants who have heard about exclusive breastfeeding	95.4	89.5	86.6	Not included
At what age should babies start eating foods in addition to breast milk? (correct answer: at 6 months)	97.6	93.9	92.2	Not included
Ways to improve diets for pregnant/lactating women	2 (1, 3)	2 (1, 3)	NA	‘Dietary knowledge’
Eat more food (more energy)	65.5	61.8	49.9	
Eat more at each meal (eat more food each day)	51.6	47.3	33.3	
Eat more frequently (eat more times each day)	51.9	50.1	35.7	
Eat more protein‐rich foods	26.9	26.4	14.7	
Eat more iron‐rich foods	13.0	12.2	5.6	
Use iodized salt when preparing meals	11.7	10.4	4.7	
% of participants who have heard of iron‐deficiency anaemia.	57.2	59.3	42.5	‘
Knowledge of iron‐rich foods	1 (0, 1)	1 (0, 1)	NA	‘Vitamin knowledge’
Organ meat (liver, kidney, heart, other)	41.3	44.1	30.4	
Flesh meats	26.0	24.9	13.9	
Insects	0.6	1.4	0.1	
Seafood (fish and shellfish)	4.4	5.2	2.0	
% of participants who have heard of vitamin A or vitamin A deficiency?	45.1	46.5	26.9	
Knowledge of vitamin A‐rich foods	1 (0, 3)	1 (0, 3)	NA	‘Vitamin knowledge’
Organ meat: Liver, kidney and heart	24.9	28.6	15.0	
Egg yolks/egg from chicken, duck, guinea fowl or other bird	29.6	28.2	16.2	
Milk, cheese, yogurt or other dairy product	26.1	26.6	13.8	
Orange‐coloured vegetables	15.0	15.9	6.6	
Other locally available vitamin A‐rich produce	14.0	13.3	5.4	
Green vegetables	20.7	20.2	9.0	
Fruits	15.3	17.1	6.8	
Red palm oil	1.4	1.4	0.4	
Ways to make porridge more nutritious for children	2 (1, 3)	2 (1, 2)	NA	‘Dietary knowledge’
Animal source foods (meat, poultry, fish, liver/organ meat, eggs, etc.)	54.6	49.1	38.8	
Pulses and nuts	50.1	44.4	34.5	
Vitamin A‐rich foods	27.0	24.8	14.2	
Green leafy vegetables	22.7	17.0	8.5	
Energy rich foods (oil and butter)	39.4	37.3	25.6	

^a^ Summary data are either presented as median with quartiles 1 and 3 (Q1, Q3) or percentages pooled across arms.

### 3.1 Does men's nutrition knowledge affect the diets of women, children, and households? Which components of knowledge have the highest effect on dietary diversity scores?

Men's dietary knowledge had higher effect on MDD‐W, and both dietary and vitamin knowledge had similar effect size on HDDS and CDDS (Tables 3 and 4). One standard deviation (SD) unit increase in men's dietary knowledge was associated with higher women's dietary diversity (0.18–0.19 food groups, see W‐models 1 and 3) even after adjusting for women's and men's education and other demographic factors. In other words, the average dietary knowledge among fathers is a score of 3.8 (mean factor scores of zero) and an increase of this knowledge by 2.0 food groups (or by 1 SD in factor scores) is associated with an increase in women's dietary diversity of 0.18–0.19 food groups. Overall, an increase in men's knowledge score of 1 SD has a comparable and additive effect as increasing women's knowledge by 1 SD.

**TABLE 3:** Mixed effects regression results from key nutrition knowledge factors on woman's dietary diversity (10 food groups, ‘W‐models’) and children's dietary diversity (seven food groups, ‘C‐models’), adjusting for demographic and household variables and village‐level clustering

Women's dietary diversity (24‐h recall)^a^	W‐model 1	W‐model 2	W‐model 3	W‐model 4	W‐model 5	W‐model 6 (interaction terms—women)	W‐model 7 (interaction terms—men)	W‐model 8 (market; subgroup *n* = 1,171)
Women's dietary knowledge	0.19^*^ [0.092, 0.29]				0.12^*^ [0.0052, 0.24]	0.15^*^ [0.0046, 0.29]	0.12^*^ [0.0059, 0.24]	0.14^*^ [0.016, 0.26]
Women's vitamin knowledge		0.13^*^ [0.035, 0.22]						
Men's dietary knowledge			0.18^*^ [0.087, 0.27]		0.11^*^ [0.0030, 0.23]	0.12^*^ [0.0058, 0.23]	0.22^*^ [0.034, 0.42]	0.12^*^ [0.00013, 0.24]
Men's vitamin knowledge				0.14^*^ [0.032, 0.24]				
Distance to market (min)								−0.0017^*^ [−0.0033, −0.00017]
Interaction terms (knowledge and education)	‐	‐	‐	‐	‐	Not significant (see Table S2)	Significant (see Figure 3 and Table S2)	‐
AIC	4,303.0	4,310.0	4,303.2	4,311.0	4,301.0	4,306.1	4,296.3	3,631.0

Children's dietary diversity (24‐h recall)^a^	C‐model 1	C‐model 2	C‐model 3	C‐model 4	C‐model 5	C‐model 6 (interaction terms—women)	C‐model 7 (interaction terms—men)	C‐model 8 (market; subgroup *n* = 613)
Women's dietary knowledge	0.19^*^ [0.018, 0.36]				0.12 [−0.077, 0.32]	0.22^b^ [−0.026, 0.47]	0.12 [−0.082, 0.32]	0.19^b^ [−0.018, 0.39]
Women's vitamin knowledge		0.033 [−0.12, 0.19]						
Men's dietary knowledge			0.19^*^ [0.018, 0.36]		0.12 [−0.077, 0.32]	0.13 [−0.070, 0.33]	0.016 [−0.37, 0.40]	0.099 [−0.11, 0.31]
Men's vitamin knowledge				0.21^*^ [0.020, 0.40]				
Distance to market (min)								−0.0017 [−0.0043, 0.00082]
Interaction term (knowledge and education)	‐	‐	‐	‐	‐	Not significant	Not significant	‐
AIC	2,594.5	2,599.2	2,594.2	2,595.6	2,594.9	2,595.9	2,598.8	2,117.2

^a^ All models were adjusted for household size, household wealth quintile, woman's age, man's age, woman's education, man's education, geographical region and kebele‐level clustering (treatment effects were not significant). Children's models additionally adjusted for age of the child. Full model results are shown in Tables S3–S5.

^b^ *P* < 0.10.

^*^ *P* < 0.05.

**TABLE 4:** Regression results of key nutrition knowledge factors on household dietary diversity scores

Household dietary diversity score^a^	H‐model 1	H‐model 2	H‐model 3	H‐model 4	H‐model 5	H‐model 6 (interaction terms—women)	H‐model 7 (interaction terms—men)	H‐model 8 (market; subgroup *n* = 1,171)
Women's dietary knowledge	0.23^*^ [0.086, 0.38]				0.13 [−0.042, 0.31]	0.28^*^ [0.065, 0.49]	0.13 [−0.048, 0.30]	0.16^b^ [−0.021, 0.35]
Women's vitamin knowledge		0.21^*^ [0.077, 0.35]						
Men's dietary knowledge			0.24^*^ [0.10, 0.38]		0.17^*^ [0.0021, 0.34]	0.18^*^ [0.013, 0.35]	0.16 [−0.12, 0.45]	0.16^b^ [−0.023, 0.34]
Men's vitamin knowledge				0.23^*^ [0.068, 0.38]				
Distance to market (minutes)								−0.0025^*^ [−0.0048, −0.00017]
Interaction term (knowledge and education)	‐	‐	‐	‐	‐	Significant (see Table S5)	Marginally significant	‐
AIC	5,428.8	5,429.0	5,427.1	5,430.6	5,426.9	5,422.6	5,425.0	4,569.9

^a^ All models adjusted for household size, household wealth quintile, woman's age, man's age, woman's education, man's education, and geographical region; adjusted for kebele‐level clustering (treatment effect were not significant). Full model results are shown in Tables S3–S5.

^b^ *P* < 0.10.

^*^ *P* < 0.05.

Households in the higher wealth quintiles had significantly higher MDD‐W by 0.29–0.35 food groups compared with the lowest two quintiles (see Tables S3–S5). Age for both men and women was not significantly associated with women's dietary diversity scores.

Men's dietary and vitamin knowledge and women's dietary knowledge was positively associated with children's dietary diversity scores (0.18, see C‐models 1–4). When both dietary knowledge from men and women of the same household were added to the model (C‐model 5), neither were significant, perhaps due to the correlation between those variables (see Figure 2d).

One SD unit in men's and women's knowledge (dietary and vitamin) was associated with increased HDDS (0.21–0.24 food groups, see H‐models 1–4 in Table 4). Men's dietary knowledge was independently associated with HDDS, even after adjusting for women's dietary knowledge and education. Age of both parents and household size was not associated with HDDS.

### 3.2 Does education modify the effect of nutrition knowledge on dietary diversity scores?

Interaction between nutrition knowledge and education varied by outcome and gender. For MDD‐W, there was no significant interaction observed between women's education and their dietary knowledge. However, significant interaction effects were observed for men's education and nutrition knowledge on MDD‐W. Among men who attended a religious school or adult literacy programmes, rather than typical formal education, higher nutrition knowledge was associated with significantly lower MDD‐W scores among women (see Figure S1). These households represent 10% of the sample population. In these same households, child dietary diversity scores were also lower by 0.39–0.40 food groups. For CDDS, there was no significant interaction between nutrition knowledge and education of either parents on children's dietary diversity scores. For HDDS, there was interaction effect observed between women's education and knowledge; households with women who had Primary 2 or religious schooling had lower HDDS (−1.27 to −0.42, see Table S5) compared to women who had no schooling.

### 3.3 How does access to market affect outcomes? Does distance to markets modify the effect of nutrition knowledge on nutrition outcomes?

Longer duration to the nearest market (in minutes) was negatively and significantly associated with MDD‐W and HDDS but not with CDDS (W‐model 8, C‐model 8 and H‐model 8). Distance to the closest market did modify the effect of women's dietary knowledge on child's dietary diversity in a very small yet significantly way, that is, women with higher dietary knowledge that are closer to a market had children with higher CDDS (results not shown). Similar results were observed for HDDS. For MDD‐W, both genders' dietary knowledge interacted significantly with distance to market (results not shown). We also noted cross‐over interaction between men's and women's dietary knowledge (*P* value = 0.05) in the subset analysis of three regions (Amhara, SNNPR and Oromia) when duration to market was included in the model. Plots showing average (model with no interaction) and interaction effects (between men's and women's dietary knowledge) are shown in Figure S2. Here, increasing knowledge among fathers was significantly associated with higher dietary diversity among children, but only among households where women had lower standardized dietary knowledge scores (factor scores below 0), which represented 50% of sample population.

### 3.4 How does nutrition knowledge affect consumption of food groups?

**FIGURE 3:**
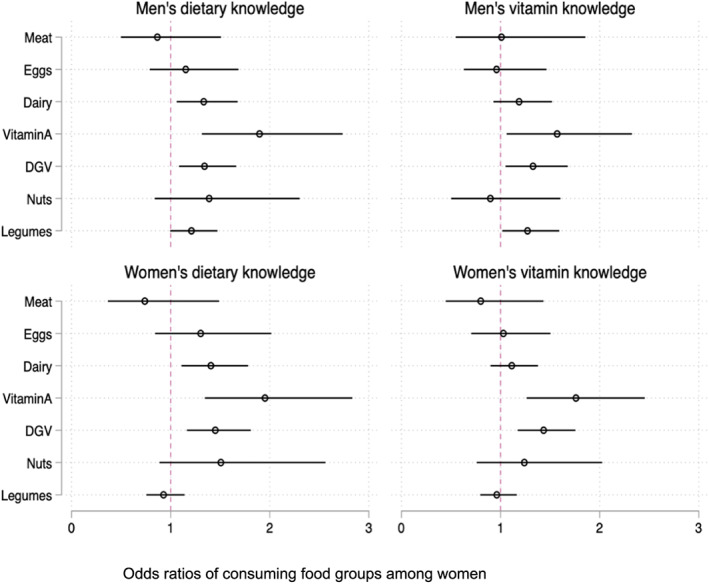
Results from mixed effects logistic regression of consuming individual food groups among women. All models adjusted for household size, household wealth quintile, women's woman's age, man's age, woman's education, man's education, region and kebele‐level clustering (treatment effect was not significant). DGV: dark green vegetables; Vitamin A: vitamin A rich produce (including both vegetables and fruits that are rich sources of vitamin A)

Overall, men's dietary knowledge was associated with significantly higher odds of women consuming dairy, vitamin A‐rich foods and dark green leafy vegetables, and the odds ratio varied for different food groups; that is, the effect of knowledge on consumption differed by food group (see Figure 3). Similar trends were observed for women's dietary knowledge. Vitamin knowledge among both men and women was associated with increased odds of women consuming vitamin A rich produce and dark green leafy vegetables.

## 4 DISCUSSION

The diets of women and children (and households generally) were very poor in this rural population in the four most populous regions of Ethiopia; only 9.4% of women and 26.7% of children met the minimum recommendation for dietary diversity. Consumption of animal source foods was low for both women and children. Knowledge of breastfeeding practices was above 80% among both men and women, possibly due to the extensive programming of Alive and Thrive in these same four regions (Menon, Rawat, & Ruel, 2013) and availability of the national health extension programme. However, knowledge on dietary practices to improve vitamin A or iron intake remained poor, with higher discordance in knowledge between men and women of the same household. Overall, men's and women's nutrition knowledge had a positive relationship with the household's dietary outcomes.

Numerous studies have illustrated that, in settings with low education, improving nutrition knowledge among women through programming can have positive impacts on children's diets (Alderman & Headey, 2017; Hirvonen et al., 2017; Onyeneke et al., 2019; Webb & Block, 2004). But very few studies have looked at how men's knowledge can improve diets. Our study's results illustrate that men's nutrition knowledge is additive to women's nutrition knowledge for improving women's and households' dietary diversity. With respect to children's dietary diversity, both men's dietary and vitamin knowledge had positive and significant associations, whereas only women's dietary knowledge has a positive and significant association. This may be because men have higher education compared with women, which may result in higher vitamin knowledge than women. We also noticed that knowledge variables of the father and mother appeared to attenuate the effect size of each otheron child dietary diversity score, possibly due to high correlation between men's and women's knowledge (*r* = 0.5; see Figure 2).

Education, wealth, and access to markets are common mediators and modifiers of women's nutrition knowledge on child nutrition outcomes (Burchi, 2010; Hirvonen et al., 2017; Onyeneke et al., 2019; Ruel et al., 1992). In this analysis, there were no interaction effects between education and knowledge for either parent on children's dietary diversity. There are several explanations for the observed results. First, most of the sampled population had a low education level; for example, 60% of mothers in this analysis had no schooling, and an additional 20% had fewer than 5 years of schooling. These results are similar to other studies (Bilal et al., 2016; Hirvonen et al., 2017; Oduor et al., 2018) where the majority of caregivers had low education. Second, substitution (and collinearity) between parents in the same household for knowledge and education attenuated the effect size of these factors individually. In samples where there is heterogeneity in education levels, women's education appears to have a larger impact than men's education on dietary diversity (Onyeneke et al., 2019; Ruel et al., 1992) and other nutrition outcomes (Alderman & Headey, 2017). A previous study in Ethiopia found that fathers' education appears to have a small positive effect (0.09 food groups) on the child's dietary diversity score (Hirvonen et al., 2017). In this study we see similar results, where fathers' education levels were not associated with CDDS, except among fathers who had religious schooling or had attended adult literacy programmes, in which case these households had lower CDDS. The percentage of men who went to religious school or literacy programmes is less than 10% (*n* = 115). In these households, men are at least 8 years older than the rest of sample population, but no other differences in demographics were observed. In these households, consumption of vitamin A rich produce is generally lower for both women and children. We also note that nutrition knowledge between men and women does seem to attenuate each other's effectsize, when both are added to the model (C‐model 5), perhaps due to the positive correlation between these variables.

In the context of NSA, there is greater emphasis on children's nutrition outcomes compared with women's outcomes. This analysis fills a research gap on women's dietary outcomes. Men's education appears to modify the effect of nutrition knowledge for women's outcomes and to a lesser extent for household outcomes, whereas women's education modifies the effect of nutrition knowledge for her own diet and the household's diet. Also, it is important to note that men's nutrition knowledge is independently associated with higher MDD‐W, even after adjusting for his and his spouse's education status and knowledge. These results are similar to a large analysis of Demographic and Health Surveys that included 69,432 mothers from 56 developing countries (Alderman & Headey, 2017). Authors found that men's education was significantly associated with higher dietary diversity among mothers (but not all women) when men have more than 7 years of schooling, whereas women's education was not significant unless she had 13+ years of schooling.

Two recent studies show that living near a market increases CDDS by one additional food group, among households with higher maternal nutrition knowledge (Hirvonen et al., 2017; Onyeneke et al., 2019). Similarly, we found a small but significant effect of time to the market on dietary diversity outcomes. We observe a smaller effect size because our models adjust for village‐level clustering, which accounts for most of the variation observed in the variable that measures households' distance to markets. Hirvonen et al. (2017) observed similar effects in their modelling approach. Regardless of the model specification, this study adds to the growing consensus that access to market is a key enabling factor. Access to market encompasses physical duration (infrastructure/transport cost), affordability, and the availability of foods. These factors are primarily driven by seasonality. A study in Kenya found that mother's nutrition knowledge predicted the seasonal changes in children's diets, suggesting that availability of foods (together with knowledge) is a necessary factor for improving diets. Similarly, in Ghana, purchased foods within community were positively associated with household dietary diversity (Christian et al., 2016). In our previous work, we have shown that availability of food from markets is seasonal in this population, thus highlighting the need for nutrition programming to be tailored for seasons and local food availability (Ambikapathi et al., 2019). Further, even if women have access to the market and have greater nutrition knowledge, they may not be the main persons who frequent the markets or the key decision makers for market purchases. Ragasa and colleagues found that giving both men and women market access advice was significantly associated with higher household dietary diversity score (0.88 food groups), compared with men alone (0.31) or women alone (0.54) (Ragasa, Aberman, & Alvarez Mingote, 2019). In their study of 3001 households in Malawi, both members (women and men) received advice on market access in only 3% of households (Ragasa et al., 2019). Future research should focus on gender‐ and culture‐appropriate strategies to improve nutrition and market access information targeted to both women and men within the same household.

There are limitations to this analysis that may affect interpretation. Breastfeeding status in the previous 24 hours was only collected at the time of child enrolment, which was at baseline; thus, we were not able to adjust for this. The median age of children was 22 months; because of their age, breasmilk might not be a substantial contribution of calories or nutrients. These associations were from cross‐sectional surveys among households with a highly seasonal food system, so caution should be exercised with regard to temporality. Finally, despite pilot‐testing of tools, it is possible that the FAO instrument measuring knowledge was not adequate for capturing nutrition knowledge for men, or generally, for this context. In this analysis, we make the assumption that the measurement error with this instrument was similar between genders, regions, and education levels.

This study is novel in that it considers men's education, age and nutritional knowledge along with women's education, age and nutrition knowledge, to examine effects on women's and children's dietary outcomes, assessing and specifically estimating the additive effects of men's characteristics for household nutrition outcomes. We also focused on specificity of exposures, such as the impact of knowledge of dietary practices on specific dietary behaviors , rather than longer‐term effects on nutritional status. Finally, we show results from multiple models to evaluate the change in coefficients of key exposures on outcomes. Although not causal, these results are useful for testing and generating new hypotheses on pathways (grey arrows in Figure 1). For example, among men and women with low education, does improving procedural dietary knowledge yield better returns than improving factual knowledge about vitamins?

Below, we have outlined key questions that still remain from this analysis. These research questions were prioritized for understanding the pathways from agriculture to nutrition outcomes and, more importantly, to add evidence for effective nutrition programmes and policies towards men's engagement in NSA: (1) the role of women's and men's empowerment dimensions (resources, autonomy, participation, time use and decision making) on moderating the effect of knowledge on dietary diversity among women and children; (2) the impact of nutrition knowledge on nutrition outcomes given the potential modifying effects of seasonality (including household changes in livelihood, expenditures, crops and livestock), market food availability and diversity; (3) water, sanitation and hygiene knowledge between members of the family, especially older siblings who aid in caregiving and household chores; (4) household and community information spillovers of knowledge and practices, and other forms of informal information flow; (5) household‐ and community‐level factors that provide opportunities to operationalize the targeted behaviours of consuming diverse food groups (wealth, education and market access are a few that have been identified); (6) key implementation characteristics and strategies of programmes to engage both men and women, which may require detailed ethnographic studies; and (7) local conceptualization of nutrition knowledge and practices and differences in these frameworks by gender, age (adolescents, school‐aged children and grandparents), and stakeholder type(food vendors, health care workers, community health workers and leaders). In future analyses, we aim to address the first three questions longitudinally, incorporating findings from a qualitative study that interviewed men and women about men's engagement in nutrition and caregiving. We invite other researchers to focus on these identified topics, especially using existing datasets from NSA programmes, to pursue the imperative and achievable target of optimal women's and children's nutrition outcomes through men's engagement..

## CONFLICTS OF INTEREST

The authors declare that they have no conflicts of interest.

## CONTRIBUTIONS

RA and NSG designed the study. WF, YB and LS led the design and implementation of the parent study that provided data for these analyses. DT, AWT and SA led the fieldwork and data collection. RA conducted the analysis and wrote the manuscript with input from all co‐authors. RA and NSG have the primary responsibility for final content. All authors read and approved the final manuscript. This paper was presented at Agriculture, Nutrition & Health (ANH) Academy Week in Accra, Ghana (2018) and won the best poster award.

## Supporting information


**Figure S1:** Average and interaction effects of men's education and dietary knowledge on women's dietary diversity. Other education group (pink line in second panel) represents religious schooling.
Figure S2: Average and interaction effects of men's and women's dietary knowledge on children's dietary score.
Table S1: Factor loadings on men's and women's knowledge (2 separate analyses).
Table 2: Regional demographics of interest from the ATONU study midline evaluation, July to August 2017, Ethiopia
Table S3: Complete results from mixed effects regression mode on women's dietary diversity score (24 h recall)
Table S4: Complete results from mixed effects regression mode on Children's dietary diversity score (24‐h recall, 7 food group.)
Table S5: Complete results from mixed effects regression mode on Household dietary diversity score (1 month recall)
