# Supplementary material for: Men's nutrition knowledge is important for women's and children's nutrition in Ethiopia
Source: Matern Child Nutr. 2020 Aug 4;17(1):e13062. doi: 10.1111/mcn.13062 (PMC7729551; doi:10.1111/mcn.13062)
Supplement: Supplementary file 1 — Figure S1: Average and interaction effects of men's education and dietary knowledge on women's dietary diversity. Other education group (pink line in second panel) represents religious schooling. Figure S2: Average and interaction effects of men's and women's dietary knowledge on children's dietary score. Table S1: Factor loadings on men's and women's knowledge (2 separate analyses). Table 2: Regional demographics of interest from the ATONU study midline evaluation, July to August 2017, Ethiopia Table S3: Complete results from mixed effects regression mode on women's dietary diversity score (24 h recall) Table S4: Complete results from mixed effects regression mode on Children's dietary diversity score (24‐h recall, 7 food group.) Table S5: Complete results from mixed effects regression mode on Household dietary diversity score (1 month recall) [file MCN-17-e13062-s001.docx]

Supplementary Figures and Tables

Supplemental figure 1: Average and interaction effects of men’s education and dietary knowledge on women’s dietary diversity. Other education group (pink line in second panel) represents religious schooling.


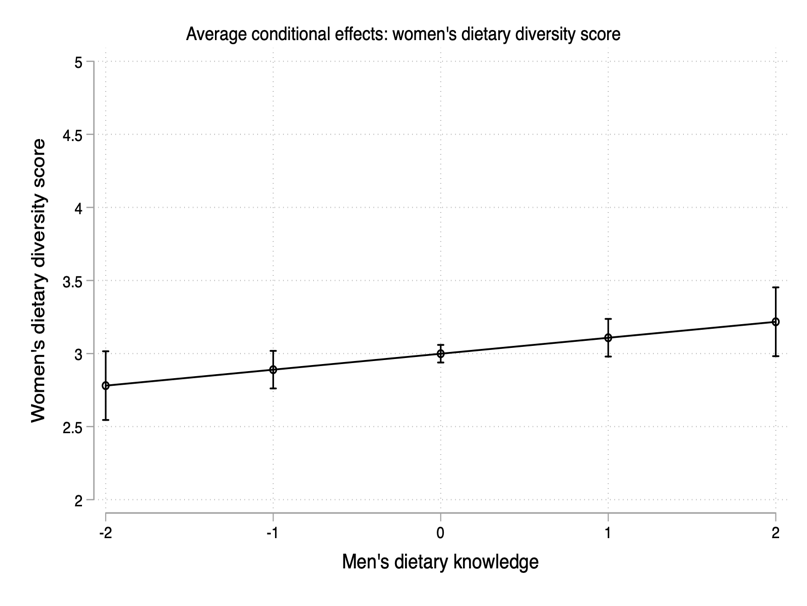

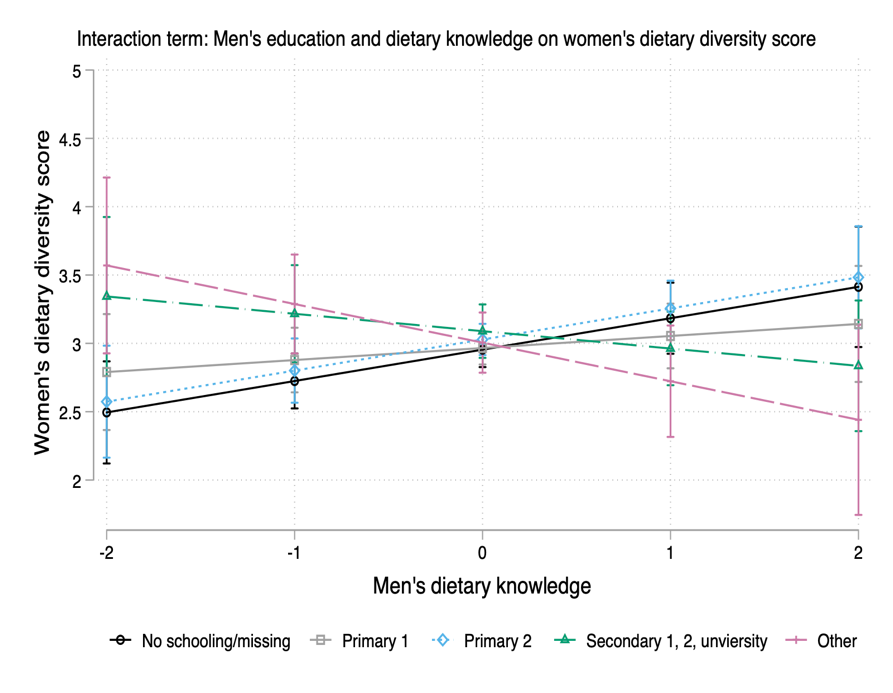


Supplement figure 2: Average and interaction effects of men’s and women’s dietary knowledge on children’s dietary score.

Low women’s dietary knowledge score

High women’s dietary knowledge score

Supplement Table 1: Factor loadings on men’s and women’s knowledge (2 separate analyses).

| Variable | Factor 1 | Factor 2 |
| --- | --- | --- |
| Men's Pregnancy dietary knowledge | 0.5523 | 0.2989 |
| Men's child dietary knowledge | 0.5694 | 0.3019 |
| Men’s iron knowledge | 0.2904 | 0.5009 |
| Men's vitamin A knowledge | 0.3825 | 0.5181 |

| Variable | Factor1 | Factor2 |
| --- | --- | --- |
| Women's Pregnancy dietary knowledge | -0.01454 | 0.34672 |
| Women's child dietary knowledge | 0.07436 | 0.36996 |
| Women’s iron knowledge | 0.37068 | -0.03625 |
| Women’s vitamin A knowledge | 0.398 | 0.06819 |

Table 2: Regional demographics of interest from the ATONU study midline evaluation, July to August 2017, Ethiopia

|  | **Amhara** | **Oromia** | **SNNPR** | **Tigray** | **Total** |
| --- | --- | --- | --- | --- | --- |
|  | **N=386** | **N=491** | **N=294** | **N=225** | **N=1,396** |
| Household Dietary Diversity Score | 5 (3,6) | 5 (4,6) | 4 (3,6) | 4 (3,5) | 4 (3,6) |
| Women's dietary diversity score - 24 hour recall | 3 (2,3) | 3 (3,4) | 2 (2,3) | 3 (2,3) | 3 (2,4) |
| % Consumption of meat food group among women - 24 hour recall | 0.5 (2) | 2.2 (11) | 1.0 (3) | 10.7 (24) | 2.9 (40) |
| % Consumption of pulses food group among women - 24 hour recall | 70.2 (271) | 56.0 (275) | 32.0 (94) | 44.0 (99) | 52.9 (739) |
| % Consumption of nuts and seeds food group among women -24 hour recall | 1.6 (6) | 6.3 (31) | 0.3 (1) | 1.3 (3) | 2.9 (41) |
| % Consumption of vitamin A rich food group among women - 24 hour recall | 3.4 (13) | 9.0 (44) | 3.7 (11) | 1.8 (4) | 5.2 (72) |
| % Consumption of dark green leafy vegetables food group among women - 24 hour recall | 22.8 (88) | 40.5 (199) | 53.4 (157) | 10.2 (23) | 33.5 (467) |
| % Consumption of eggs food group among women - 24 hour recall | 0.3 (1) | 6.1 (30) | 3.7 (11) | 21.3 (48) | 6.4 (90) |
| Women meeting minimum dietary diversity (binary, <5 food groups) - 24 hour recall | 3.1 (12) | 17.1 (84) | 8.8 (26) | 4.4 (10) | 9.5 (132) |
|  |  |  |  |  |  |
| Children's dietary diversity score with 7 food groups (original indicator) | 3 (2,3) | 3 (2,4) | 2 (1,3) | 2 (0,3) | 3 (1,3) |
| Children meeting minimum dietary diversity (binary, <4 food groups) | 22.2 (42) | 35.3 (97) | 19.5 (29) | 8.9 (11) | 24.3 (179) |
| % Consumption of flesh foods group among children | 1.1 (2) | 1.5 (4) | 1.3 (2) | 2.4 (3) | 1.5 (11) |
| % Consumption of pulses/nuts food group among children | 52.4 (99) | 41.8 (115) | 20.1 (30) | 22.0 (27) | 36.8 (271) |
| % Consumption of vitamin A rich vegetables and fruits food group among children - | 14.8 (28) | 35.6 (98) | 43.6 (65) | 5.7 (7) | 26.9 (198) |
| % Consumption of other fruits/vegetables food group among children | 56.1 (106) | 69.1 (190) | 37.6 (56) | 39.0 (48) | 54.3 (400) |
| % Consumption of fats food group among children | 40.7 (77) | 48.0 (132) | 33.6 (50) | 41.5 (51) | 42.1 (310) |
| % Consumption of eggs food group among children | 7.9 (15) | 12.7 (35) | 5.4 (8) | 16.3 (20) | 10.6 (78) |
|  |  |  |  |  |  |
| Maternal age (years) | 35 (30,40) | 32 (26,39) | 34 (30,38) | 35 (27,40) | 34 (28,39) |
| Men's age (years) | 42 (36,50) | 40 (32,46) | 40 (35,48) | 44 (38,52) | 40 (35,48) |
| Children’s age (months) | 22 (13,30) | 22 (14,34) | 24 (15,32) | 21 (12,30) | 22 (13,32) |
|  |  |  |  |  |  |
| Women's education |  |  |  |  |  |
| No schooling/missing | 69.9 (270) | 54.2 (266) | 54.8 (161) | 60.0 (135) | 59.6 (832) |
| Primary 1 | 10.9 (42) | 24.6 (121) | 21.4 (63) | 19.1 (43) | 19.3 (269) |
| Primary 2 | 9.6 (37) | 14.1 (69) | 16.7 (49) | 15.1 (34) | 13.5 (189) |
| Secondary 1, 2, university | 4.1 (16) | 4.3 (21) | 4.8 (14) | 4.9 (11) | 4.4 (62) |
| Other | 5.4 (21) | 2.9 (14) | 2.4 (7) | 0.9 (2) | 3.2 (44) |
| Men's education |  |  |  |  |  |
| No schooling/missing | 35.0 (135) | 21.6 (106) | 20.7 (61) | 30.2 (68) | 26.5 (370) |
| Primary 1 | 18.1 (70) | 29.3 (144) | 24.5 (72) | 25.8 (58) | 24.6 (344) |
| Primary 2 | 19.4 (75) | 31.4 (154) | 37.8 (111) | 26.7 (60) | 28.7 (400) |
| Secondary 1, 2, university | 8.3 (32) | 15.1 (74) | 16.0 (47) | 6.2 (14) | 12.0 (167) |
| Other | 19.2 (74) | 2.6 (13) | 1.0 (3) | 11.1 (25) | 8.2 (115) |
| % Access to improved Water | 73.6 (284) | 83.5 (409) | 74.4 (218) | 86.2 (194) | 79.3 (1,105) |
| % Access to improved Sanitation | 35.2 (136) | 28.1 (138) | 58.7 (172) | 8.4 (19) | 33.3 (465) |
| % Land owned (timad) | 4 (2,6) | 6 (3,10) | 2 (1,4) | 3 (2,4) | 4 (2,6) |
| Total number of HH members | 7 (5,8) | 7 (5,8) | 7 (5,8) | 6 (5,8) | 7 (5,8) |
| Distance to the closest market (minutes) | 60 (30,100) | 40 (25,60) | 30 (20,60) |  | 45 (30,60) |
| Household Food Insecurity Access (FIA) |  |  |  |  |  |
| Food secure | 53.6 (207) | 47.7 (234) | 36.4 (107) | 76.0 (171) | 51.5 (719) |
| Mildly FIA | 8.3 (32) | 10.2 (50) | 12.2 (36) | 4.9 (11) | 9.2 (129) |
| Moderate FIA | 21.8 (84) | 19.8 (97) | 31.6 (93) | 6.2 (14) | 20.6 (288) |
| Severe FIA | 16.3 (63) | 22.4 (110) | 19.7 (58) | 12.9 (29) | 18.6 (260) |

Supplement Table 3: Complete results from mixed effects regression mode on women’s dietary diversity score (24 hour recall)

| Women’s Dietary Diversity (24-hour recall) | W-Model 1 | W-Model 2 | W-Model 3 | W-Model 4 | W-Model 5 | W- Model 6 (Int. with women’s education) | W- Model 7 (Int. with men’s education) | W- Model 8 (Market; Sub group n =1171) |
| --- | --- | --- | --- | --- | --- | --- | --- | --- |
| Women's dietary knowledge | 0.19^*^ |  |  |  | 0.12^*^ | 0.15^*^ | 0.12^*^ | 0.14^*^ |
|  | [0.092,0.29] |  |  |  | [0.0052,0.24] | [0.0046,0.29] | [0.0059,0.24] | [0.016,0.26] |
|  |  |  |  |  |  |  |  |  |
| Women's vitamin knowledge |  | 0.13^*^ |  |  |  |  |  |  |
|  |  | [0.035,0.22] |  |  |  |  |  |  |
|  |  |  |  |  |  |  |  |  |
| Men's dietary knowledge |  |  | 0.18^*^ |  | 0.11^*^ | 0.12^*^ | 0.22^*^ | 0.12^*^ |
|  |  |  | [0.087,0.27] |  | [0.0030,0.23] | [0.0058,0.23] | [0.034,0.42] | [0.00013,0.24] |
|  |  |  |  |  |  |  |  |  |
| Men's vitamin knowledge |  |  |  | 0.14^*^ |  |  |  |  |
|  |  |  |  | [0.032,0.24] |  |  |  |  |
|  |  |  |  |  |  |  |  |  |
| Amhara | 0 | 0 | 0 | 0 | 0 | 0 | 0 | 0 |
|  | [0,0] | [0,0] | [0,0] | [0,0] | [0,0] | [0,0] | [0,0] | [0,0] |
|  |  |  |  |  |  |  |  |  |
| Oromia | 0.64^*^ | 0.65^*^ | 0.63^*^ | 0.64^*^ | 0.63^*^ | 0.63^*^ | 0.63^*^ | 0.57^*^ |
|  | [0.40,0.89] | [0.40,0.90] | [0.38,0.89] | [0.39,0.90] | [0.38,0.88] | [0.38,0.87] | [0.38,0.88] | [0.31,0.82] |
|  |  |  |  |  |  |  |  |  |
| SNNPR | -0.24^+^ | -0.25^+^ | -0.24^+^ | -0.25^+^ | -0.24^+^ | -0.24^+^ | -0.24^+^ | -0.31^*^ |
|  | [-0.51,0.032] | [-0.53,0.024] | [-0.52,0.037] | [-0.53,0.028] | [-0.52,0.030] | [-0.51,0.032] | [-0.52,0.028] | [-0.60,-0.028] |
|  |  |  |  |  |  |  |  |  |
| Tigray | 0.18 | 0.047 | 0.081 | 0.049 | 0.14 | 0.14 | 0.13 |  |
|  | [-0.10,0.46] | [-0.24,0.34] | [-0.21,0.37] | [-0.24,0.34] | [-0.15,0.42] | [-0.15,0.42] | [-0.16,0.42] |  |
|  |  |  |  |  |  |  |  |  |
| Wealth quintile 1 | 0 | 0 | 0 | 0 | 0 | 0 | 0 | 0 |
|  | [0,0] | [0,0] | [0,0] | [0,0] | [0,0] | [0,0] | [0,0] | [0,0] |
|  |  |  |  |  |  |  |  |  |
| Wealth quintile 2 | 0.11 | 0.096 | 0.099 | 0.099 | 0.11 | 0.11 | 0.10 | 0.21^+^ |
|  | [-0.078,0.30] | [-0.095,0.29] | [-0.092,0.29] | [-0.092,0.29] | [-0.081,0.30] | [-0.079,0.30] | [-0.086,0.29] | [-0.018,0.44] |
|  |  |  |  |  |  |  |  |  |
| Wealth quintile 3 | 0.21^*^ | 0.19^+^ | 0.20^+^ | 0.19^+^ | 0.21^*^ | 0.21^*^ | 0.20^+^ | 0.31^*^ |
|  | [0.0084,0.42] | [-0.011,0.40] | [-0.0070,0.40] | [-0.019,0.39] | [0.0058,0.41] | [0.0053,0.41] | [-0.00072,0.41] | [0.071,0.54] |
|  |  |  |  |  |  |  |  |  |
| Wealth quintile 4 | 0.19^+^ | 0.18 | 0.19^+^ | 0.19^+^ | 0.19^+^ | 0.19^+^ | 0.19^+^ | 0.24^+^ |
|  | [-0.029,0.42] | [-0.043,0.40] | [-0.034,0.41] | [-0.036,0.41] | [-0.028,0.42] | [-0.030,0.41] | [-0.027,0.42] | [-0.0043,0.49] |
|  |  |  |  |  |  |  |  |  |
| Wealth quintile 5 | 0.29^*^ | 0.28^*^ | 0.29^*^ | 0.28^*^ | 0.29^*^ | 0.29^*^ | 0.29^*^ | 0.35^*^ |
|  | [0.078,0.50] | [0.067,0.49] | [0.077,0.50] | [0.066,0.49] | [0.083,0.50] | [0.084,0.50] | [0.083,0.50] | [0.11,0.59] |
|  |  |  |  |  |  |  |  |  |
| Women's age | -0.0027 | -0.0027 | -0.0037 | -0.0031 | -0.0034 | -0.0038 | -0.0041 | -0.0044 |
|  | [-0.014,0.0083] | [-0.014,0.0083] | [-0.015,0.0073] | [-0.014,0.0079] | [-0.014,0.0076] | [-0.015,0.0072] | [-0.015,0.0069] | [-0.017,0.0081] |
|  |  |  |  |  |  |  |  |  |
| Men's age | -0.000015 | -0.000032 | 0.00068 | 0.00027 | 0.00049 | 0.00070 | 0.00069 | 0.00046 |
|  | [-0.0078,0.0078] | [-0.0078,0.0078] | [-0.0071,0.0085] | [-0.0075,0.0081] | [-0.0073,0.0083] | [-0.0071,0.0085] | [-0.0071,0.0085] | [-0.0083,0.0092] |
|  |  |  |  |  |  |  |  |  |
| Total number of HH members | 0.028^+^ | 0.029^+^ | 0.027^+^ | 0.027^+^ | 0.028^+^ | 0.029^+^ | 0.028^+^ | 0.029^+^ |
|  | [-0.0031,0.060] | [-0.0028,0.060] | [-0.0045,0.059] | [-0.0044,0.059] | [-0.0039,0.059] | [-0.0027,0.060] | [-0.0037,0.059] | [-0.0050,0.063] |
|  |  |  |  |  |  |  |  |  |
| No schooling/missing | 0 | 0 | 0 | 0 | 0 | 0 | 0 | 0 |
|  | [0,0] | [0,0] | [0,0] | [0,0] | [0,0] | [0,0] | [0,0] | [0,0] |
|  |  |  |  |  |  |  |  |  |
| Primary 1 | -0.045 | -0.039 | -0.032 | -0.031 | -0.042 | -0.045 | -0.035 | 0.053 |
|  | [-0.21,0.12] | [-0.20,0.12] | [-0.19,0.13] | [-0.19,0.13] | [-0.20,0.12] | [-0.21,0.12] | [-0.20,0.13] | [-0.13,0.23] |
|  |  |  |  |  |  |  |  |  |
| Primary 2 | 0.11 | 0.12 | 0.13 | 0.13 | 0.12 | 0.12 | 0.13 | 0.10 |
|  | [-0.083,0.31] | [-0.080,0.32] | [-0.064,0.33] | [-0.069,0.33] | [-0.074,0.32] | [-0.076,0.32] | [-0.070,0.32] | [-0.12,0.32] |
|  |  |  |  |  |  |  |  |  |
| Secondary 1, 2, university | 0.093 | 0.11 | 0.12 | 0.13 | 0.091 | 0.064 | 0.10 | -0.066 |
|  | [-0.23,0.42] | [-0.22,0.43] | [-0.21,0.44] | [-0.19,0.46] | [-0.23,0.41] | [-0.27,0.40] | [-0.22,0.43] | [-0.43,0.29] |
|  |  |  |  |  |  |  |  |  |
| Other | 0.26 | 0.23 | 0.26 | 0.26 | 0.26 | 0.28 | 0.25 | 0.29 |
|  | [-0.085,0.61] | [-0.11,0.58] | [-0.091,0.60] | [-0.085,0.61] | [-0.090,0.60] | [-0.070,0.62] | [-0.097,0.59] | [-0.069,0.65] |
|  |  |  |  |  |  |  |  |  |
| No schooling/missing | 0 | 0 | 0 | 0 | 0 | 0 | 0 | 0 |
|  | [0,0] | [0,0] | [0,0] | [0,0] | [0,0] | [0,0] | [0,0] | [0,0] |
|  |  |  |  |  |  |  |  |  |
| Primary 1 | 0.022 | 0.032 | 0.013 | 0.027 | 0.0092 | 0.011 | -0.013 | -0.048 |
|  | [-0.15,0.19] | [-0.14,0.20] | [-0.16,0.18] | [-0.14,0.20] | [-0.16,0.18] | [-0.16,0.18] | [-0.19,0.16] | [-0.24,0.14] |
|  |  |  |  |  |  |  |  |  |
| Primary 2 | 0.12 | 0.12 | 0.087 | 0.10 | 0.094 | 0.094 | 0.056 | 0.041 |
|  | [-0.053,0.29] | [-0.051,0.30] | [-0.088,0.26] | [-0.071,0.28] | [-0.080,0.27] | [-0.081,0.27] | [-0.12,0.23] | [-0.15,0.23] |
|  |  |  |  |  |  |  |  |  |
| Secondary 1, 2, university | 0.14 | 0.14 | 0.079 | 0.096 | 0.097 | 0.100 | 0.12 | 0.10 |
|  | [-0.098,0.37] | [-0.10,0.37] | [-0.16,0.32] | [-0.14,0.34] | [-0.14,0.34] | [-0.14,0.34] | [-0.12,0.36] | [-0.16,0.36] |
|  |  |  |  |  |  |  |  |  |
| Other | 0.12 | 0.13 | 0.12 | 0.13 | 0.11 | 0.11 | 0.049 | -0.010 |
|  | [-0.13,0.36] | [-0.11,0.37] | [-0.12,0.36] | [-0.12,0.37] | [-0.13,0.35] | [-0.13,0.35] | [-0.20,0.30] | [-0.29,0.27] |
|  |  |  |  |  |  |  |  |  |
| No schooling/missing # Women's dietary knowledge |  |  |  |  |  | 0 |  |  |
|  |  |  |  |  |  | [0,0] |  |  |
|  |  |  |  |  |  |  |  |  |
| Primary 1 # Women's dietary knowledge |  |  |  |  |  | -0.011 |  |  |
|  |  |  |  |  |  | [-0.25,0.23] |  |  |
|  |  |  |  |  |  |  |  |  |
| Primary 2 # Women's dietary knowledge |  |  |  |  |  | -0.092 |  |  |
|  |  |  |  |  |  | [-0.37,0.19] |  |  |
|  |  |  |  |  |  |  |  |  |
| Secondary 1, 2, university # Women's dietary knowledge |  |  |  |  |  | 0.066 |  |  |
|  |  |  |  |  |  | [-0.37,0.51] |  |  |
|  |  |  |  |  |  |  |  |  |
| Other # Women's dietary knowledge |  |  |  |  |  | -0.43 |  |  |
|  |  |  |  |  |  | [-0.98,0.12] |  |  |
|  |  |  |  |  |  |  |  |  |
| No schooling/missing # Men's dietary knowledge |  |  |  |  |  |  | 0 |  |
|  |  |  |  |  |  |  | [0,0] |  |
|  |  |  |  |  |  |  |  |  |
| Primary 1 # Men's dietary knowledge |  |  |  |  |  |  | -0.095 |  |
|  |  |  |  |  |  |  | [-0.36,0.17] |  |
|  |  |  |  |  |  |  |  |  |
| Primary 2 # Men's dietary knowledge |  |  |  |  |  |  | 0.015 |  |
|  |  |  |  |  |  |  | [-0.24,0.27] |  |
|  |  |  |  |  |  |  |  |  |
| Secondary 1, 2, university # Men's dietary knowledge |  |  |  |  |  |  | -0.33^*^ |  |
|  |  |  |  |  |  |  | [-0.63,-0.033] |  |
|  |  |  |  |  |  |  |  |  |
| Other # Men's dietary knowledge |  |  |  |  |  |  | -0.49^*^ |  |
|  |  |  |  |  |  |  | [-0.84,-0.13] |  |
|  |  |  |  |  |  |  |  |  |
| Distance to the market (minutes) |  |  |  |  |  |  |  | -0.0017^*^ |
|  |  |  |  |  |  |  |  | [-0.0033,-0.00017] |
|  |  |  |  |  |  |  |  |  |
| Constant | 2.44^*^ | 2.47^*^ | 2.49^*^ | 2.49^*^ | 2.48^*^ | 2.47^*^ | 2.51^*^ | 2.60^*^ |
|  | [2.03,2.86] | [2.05,2.89] | [2.08,2.91] | [2.07,2.91] | [2.06,2.89] | [2.06,2.89] | [2.10,2.93] | [2.14,3.07] |
|  | [0.051,0.13] | [0.052,0.13] | [0.050,0.13] | [0.053,0.13] | [0.049,0.12] | [0.048,0.12] | [0.045,0.12] | [0.051,0.13] |
| *AIC* | 4303.0 | 4310.0 | 4303.2 | 4311.0 | 4301.0 | 4306.1 | 4296.3 | 3631.0 |
| *BIC* | 4418.3 | 4425.3 | 4418.4 | 4426.2 | 4421.5 | 4447.6 | 4437.8 | 3747.5 |
| Log likelihood | -2129.5 | -2133.0 | -2129.6 | -2133.5 | -2127.5 | -2126.1 | -2121.1 | -1792.5 |
| Chi-squared | 99.9 | 89.5 | 97.3 | 88.4 | 103.0 | 106.4 | 117.1 | 101.2 |

95% confidence intervals in brackets

^+^ *p* < 0.10, ^*^ *p* < 0.05

Supplement Table 4: Complete results from mixed effects regression mode on Children’s dietary diversity score (24-hour recall, 7 food group.)

| Children’s Dietary Diversity (24-hour recall) | C-model 1 | C-Model 2 | C-Model 3 | C-Model 4 | C-Model 5 | C- Model 6 (Int. with women’s education) | C- Model 7 (Int. with men’s education) | C- Model 8 (Market; Sub group n =1171) |
| --- | --- | --- | --- | --- | --- | --- | --- | --- |
|  |  |  |  |  |  |  |  |  |
| Women's dietary knowledge | 0.19^*^ |  |  |  | 0.12 | 0.22^+^ | 0.12 | 0.19^+^ |
|  | [0.018,0.36] |  |  |  | [-0.077,0.32] | [-0.026,0.47] | [-0.082,0.32] | [-0.018,0.39] |
|  |  |  |  |  |  |  |  |  |
| Women's vitamin knowledge |  | 0.033 |  |  |  |  |  |  |
|  |  | [-0.12,0.19] |  |  |  |  |  |  |
|  |  |  |  |  |  |  |  |  |
| Men's dietary knowledge |  |  | 0.19^*^ |  | 0.12 | 0.13 | 0.016 | 0.099 |
|  |  |  | [0.018,0.36] |  | [-0.077,0.32] | [-0.070,0.33] | [-0.37,0.40] | [-0.11,0.31] |
|  |  |  |  |  |  |  |  |  |
| Men's vitamin knowledge |  |  |  | 0.21^*^ |  |  |  |  |
|  |  |  |  | [0.020,0.40] |  |  |  |  |
|  |  |  |  |  |  |  |  |  |
| Amhara | 0 | 0 | 0 | 0 | 0 | 0 | 0 | 0 |
|  | [0,0] | [0,0] | [0,0] | [0,0] | [0,0] | [0,0] | [0,0] | [0,0] |
|  |  |  |  |  |  |  |  |  |
| Oromia | 0.40 | 0.43 | 0.40 | 0.39 | 0.39 | 0.37 | 0.40 | 0.27 |
|  | [-0.15,0.96] | [-0.13,0.99] | [-0.16,0.96] | [-0.18,0.95] | [-0.17,0.95] | [-0.20,0.93] | [-0.15,0.96] | [-0.11,0.64] |
|  |  |  |  |  |  |  |  |  |
| SNNPR | -0.61^*^ | -0.60^+^ | -0.61^+^ | -0.64^*^ | -0.61^*^ | -0.63^*^ | -0.60^+^ | -0.75^*^ |
|  | [-1.23,-0.0016] | [-1.22,0.011] | [-1.22,0.0087] | [-1.26,-0.025] | [-1.23,-0.00084] | [-1.24,-0.012] | [-1.22,0.0074] | [-1.18,-0.33] |
|  |  |  |  |  |  |  |  |  |
| Tigray | -0.83^*^ | -0.92^*^ | -0.93^*^ | -1.00^*^ | -0.88^*^ | -0.90^*^ | -0.86^*^ |  |
|  | [-1.47,-0.20] | [-1.56,-0.28] | [-1.57,-0.29] | [-1.65,-0.36] | [-1.52,-0.23] | [-1.55,-0.26] | [-1.51,-0.22] | (not sampled) |
|  |  |  |  |  |  |  |  |  |
| Wealth quintile 1 | 0 | 0 | 0 | 0 | 0 | 0 | 0 | 0 |
|  | [0,0] | [0,0] | [0,0] | [0,0] | [0,0] | [0,0] | [0,0] | [0,0] |
|  |  |  |  |  |  |  |  |  |
| Wealth quintile 2 | 0.22 | 0.20 | 0.21 | 0.22 | 0.22 | 0.21 | 0.22 | 0.55^*^ |
|  | [-0.12,0.56] | [-0.14,0.54] | [-0.12,0.55] | [-0.12,0.56] | [-0.12,0.56] | [-0.13,0.55] | [-0.12,0.56] | [0.15,0.95] |
|  |  |  |  |  |  |  |  |  |
| Wealth quintile 3 | 0.25 | 0.22 | 0.23 | 0.23 | 0.25 | 0.25 | 0.24 | 0.51^*^ |
|  | [-0.12,0.62] | [-0.15,0.59] | [-0.13,0.60] | [-0.14,0.59] | [-0.12,0.62] | [-0.12,0.62] | [-0.12,0.61] | [0.11,0.90] |
|  |  |  |  |  |  |  |  |  |
| Wealth quintile 4 | 0.36^+^ | 0.35^+^ | 0.36^+^ | 0.37^+^ | 0.36^+^ | 0.35^+^ | 0.35^+^ | 0.66^*^ |
|  | [-0.045,0.76] | [-0.057,0.75] | [-0.047,0.76] | [-0.034,0.77] | [-0.041,0.76] | [-0.054,0.75] | [-0.052,0.75] | [0.23,1.08] |
|  |  |  |  |  |  |  |  |  |
| Wealth quintile 5 | 0.26 | 0.24 | 0.27 | 0.26 | 0.27 | 0.25 | 0.27 | 0.56^*^ |
|  | [-0.13,0.65] | [-0.15,0.63] | [-0.12,0.66] | [-0.13,0.64] | [-0.12,0.66] | [-0.13,0.64] | [-0.12,0.66] | [0.15,0.97] |
|  |  |  |  |  |  |  |  |  |
| Maternal age | -0.0057 | -0.0054 | -0.0060 | -0.0059 | -0.0060 | -0.0073 | -0.0073 | -0.0040 |
|  | [-0.031,0.019] | [-0.031,0.020] | [-0.031,0.019] | [-0.031,0.019] | [-0.031,0.019] | [-0.032,0.018] | [-0.032,0.018] | [-0.033,0.025] |
|  |  |  |  |  |  |  |  |  |
| Men's age | 0.0029 | 0.0028 | 0.0035 | 0.0034 | 0.0034 | 0.0054 | 0.0037 | -0.0042 |
|  | [-0.013,0.019] | [-0.013,0.019] | [-0.012,0.019] | [-0.012,0.019] | [-0.012,0.019] | [-0.010,0.021] | [-0.012,0.019] | [-0.022,0.014] |
|  |  |  |  |  |  |  |  |  |
| Total number of HH members | 0.057^+^ | 0.055 | 0.054 | 0.055 | 0.056 | 0.058^+^ | 0.064^+^ | 0.095^*^ |
|  | [-0.011,0.13] | [-0.013,0.12] | [-0.014,0.12] | [-0.013,0.12] | [-0.012,0.12] | [-0.0095,0.13] | [-0.0040,0.13] | [0.023,0.17] |
|  |  |  |  |  |  |  |  |  |
| No schooling/missing | 0 | 0 | 0 | 0 | 0 | 0 | 0 | 0 |
|  | [0,0] | [0,0] | [0,0] | [0,0] | [0,0] | [0,0] | [0,0] | [0,0] |
|  |  |  |  |  |  |  |  |  |
| Primary 1 | -0.15 | -0.15 | -0.15 | -0.15 | -0.15 | -0.14 | -0.15 | -0.087 |
|  | [-0.43,0.14] | [-0.43,0.14] | [-0.43,0.14] | [-0.43,0.13] | [-0.43,0.14] | [-0.42,0.14] | [-0.43,0.13] | [-0.39,0.22] |
|  |  |  |  |  |  |  |  |  |
| Primary 2 | 0.0062 | 0.020 | 0.021 | 0.011 | 0.0100 | 0.020 | 0.011 | -0.025 |
|  | [-0.34,0.35] | [-0.33,0.37] | [-0.33,0.37] | [-0.34,0.36] | [-0.34,0.36] | [-0.33,0.37] | [-0.34,0.36] | [-0.40,0.35] |
|  |  |  |  |  |  |  |  |  |
| Secondary 1, 2, university | 0.17 | 0.20 | 0.15 | 0.16 | 0.15 | 0.063 | 0.13 | 0.047 |
|  | [-0.39,0.73] | [-0.37,0.76] | [-0.41,0.71] | [-0.40,0.72] | [-0.41,0.71] | [-0.51,0.64] | [-0.43,0.69] | [-0.55,0.65] |
|  |  |  |  |  |  |  |  |  |
| Other | 0.23 | 0.25 | 0.19 | 0.21 | 0.19 | 0.36 | 0.22 | 0.50 |
|  | [-0.64,1.10] | [-0.62,1.13] | [-0.68,1.07] | [-0.66,1.08] | [-0.68,1.06] | [-0.56,1.28] | [-0.65,1.09] | [-0.41,1.41] |
|  |  |  |  |  |  |  |  |  |
| No schooling/missing | 0 | 0 | 0 | 0 | 0 | 0 | 0 | 0 |
|  | [0,0] | [0,0] | [0,0] | [0,0] | [0,0] | [0,0] | [0,0] | [0,0] |
|  |  |  |  |  |  |  |  |  |
| Primary 1 | -0.036 | -0.020 | -0.036 | -0.038 | -0.042 | -0.027 | -0.037 | -0.090 |
|  | [-0.35,0.28] | [-0.34,0.30] | [-0.35,0.28] | [-0.36,0.28] | [-0.36,0.28] | [-0.34,0.29] | [-0.36,0.28] | [-0.43,0.25] |
|  |  |  |  |  |  |  |  |  |
| Primary 2 | 0.099 | 0.11 | 0.071 | 0.062 | 0.077 | 0.064 | 0.068 | 0.055 |
|  | [-0.21,0.40] | [-0.20,0.42] | [-0.24,0.38] | [-0.25,0.37] | [-0.23,0.38] | [-0.24,0.37] | [-0.24,0.38] | [-0.28,0.39] |
|  |  |  |  |  |  |  |  |  |
| Secondary 1, 2, university | -0.044 | -0.036 | -0.11 | -0.12 | -0.087 | -0.081 | -0.090 | 0.026 |
|  | [-0.46,0.37] | [-0.45,0.38] | [-0.52,0.31] | [-0.54,0.30] | [-0.51,0.33] | [-0.50,0.34] | [-0.52,0.34] | [-0.42,0.47] |
|  |  |  |  |  |  |  |  |  |
| Other | -0.40^+^ | -0.39 | -0.41^+^ | -0.42^+^ | -0.41^+^ | -0.41^+^ | -0.40^+^ | -0.36 |
|  | [-0.87,0.067] | [-0.86,0.083] | [-0.87,0.062] | [-0.89,0.049] | [-0.88,0.058] | [-0.87,0.060] | [-0.88,0.072] | [-0.91,0.20] |
|  |  |  |  |  |  |  |  |  |
| 7-9 mon | 0 | 0 | 0 | 0 | 0 | 0 | 0 | 0 |
|  | [0,0] | [0,0] | [0,0] | [0,0] | [0,0] | [0,0] | [0,0] | [0,0] |
|  |  |  |  |  |  |  |  |  |
| 10-12 mon | 0.68^*^ | 0.69^*^ | 0.69^*^ | 0.68^*^ | 0.68^*^ | 0.66^*^ | 0.67^*^ | 0.88^*^ |
|  | [0.13,1.23] | [0.14,1.24] | [0.14,1.24] | [0.12,1.23] | [0.13,1.23] | [0.11,1.21] | [0.12,1.22] | [0.28,1.47] |
|  |  |  |  |  |  |  |  |  |
| 13-18 mon | 0.74^*^ | 0.75^*^ | 0.74^*^ | 0.75^*^ | 0.73^*^ | 0.70^*^ | 0.72^*^ | 1.01^*^ |
|  | [0.24,1.24] | [0.25,1.25] | [0.24,1.23] | [0.25,1.25] | [0.24,1.23] | [0.20,1.19] | [0.23,1.22] | [0.48,1.55] |
|  |  |  |  |  |  |  |  |  |
| 19-24 mon | 1.03^*^ | 1.05^*^ | 1.03^*^ | 1.03^*^ | 1.02^*^ | 1.00^*^ | 1.04^*^ | 1.28^*^ |
|  | [0.54,1.52] | [0.56,1.54] | [0.54,1.52] | [0.54,1.52] | [0.53,1.51] | [0.51,1.49] | [0.55,1.53] | [0.76,1.81] |
|  |  |  |  |  |  |  |  |  |
| 25-36 mon | 1.18^*^ | 1.18^*^ | 1.17^*^ | 1.17^*^ | 1.17^*^ | 1.15^*^ | 1.17^*^ | 1.46^*^ |
|  | [0.70,1.65] | [0.71,1.66] | [0.70,1.64] | [0.70,1.64] | [0.70,1.64] | [0.68,1.62] | [0.70,1.64] | [0.96,1.96] |
|  |  |  |  |  |  |  |  |  |
| 37-48 mon | 1.24^*^ | 1.26^*^ | 1.26^*^ | 1.26^*^ | 1.25^*^ | 1.21^*^ | 1.26^*^ | 1.57^*^ |
|  | [0.73,1.75] | [0.75,1.77] | [0.75,1.76] | [0.76,1.77] | [0.74,1.75] | [0.70,1.72] | [0.75,1.76] | [1.02,2.11] |
|  |  |  |  |  |  |  |  |  |
| 61+mon | -0.71 | -0.72 | -0.72 | -0.71 | -0.71 | -0.76 | -0.70 |  |
|  | [-2.65,1.24] | [-2.67,1.23] | [-2.67,1.22] | [-2.65,1.24] | [-2.65,1.24] | [-2.70,1.17] | [-2.63,1.24] |  |
|  |  |  |  |  |  |  |  |  |
| No schooling/missing # Women's dietary knowledge |  |  |  |  |  | 0 |  |  |
|  |  |  |  |  |  | [0,0] |  |  |
|  |  |  |  |  |  |  |  |  |
| Primary 1 # Women's dietary knowledge |  |  |  |  |  | -0.34 |  |  |
|  |  |  |  |  |  | [-0.75,0.077] |  |  |
|  |  |  |  |  |  |  |  |  |
| Primary 2 # Women's dietary knowledge |  |  |  |  |  | -0.14 |  |  |
|  |  |  |  |  |  | [-0.59,0.31] |  |  |
|  |  |  |  |  |  |  |  |  |
| Secondary 1, 2, university # Women's dietary knowledge |  |  |  |  |  | 0.24 |  |  |
|  |  |  |  |  |  | [-0.52,1.00] |  |  |
|  |  |  |  |  |  |  |  |  |
| Other # Women's dietary knowledge |  |  |  |  |  | -1.02 |  |  |
|  |  |  |  |  |  | [-2.64,0.59] |  |  |
|  |  |  |  |  |  |  |  |  |
| No schooling/missing # Men's dietary knowledge |  |  |  |  |  |  | 0 |  |
|  |  |  |  |  |  |  | [0,0] |  |
|  |  |  |  |  |  |  |  |  |
| Primary 1 # Men's dietary knowledge |  |  |  |  |  |  | -0.10 |  |
|  |  |  |  |  |  |  | [-0.61,0.40] |  |
|  |  |  |  |  |  |  |  |  |
| Primary 2 # Men's dietary knowledge |  |  |  |  |  |  | 0.31 |  |
|  |  |  |  |  |  |  | [-0.17,0.79] |  |
|  |  |  |  |  |  |  |  |  |
| Secondary 1, 2, university # Men's dietary knowledge |  |  |  |  |  |  | 0.18 |  |
|  |  |  |  |  |  |  | [-0.36,0.72] |  |
|  |  |  |  |  |  |  |  |  |
| Other # Men's dietary knowledge |  |  |  |  |  |  | 0.028 |  |
|  |  |  |  |  |  |  | [-0.70,0.75] |  |
|  |  |  |  |  |  |  |  |  |
| Distance to the market (minutes) |  |  |  |  |  |  |  | -0.0017 |
|  |  |  |  |  |  |  |  | [-0.0043,0.00082] |
|  |  |  |  |  |  |  |  |  |
| R^2^ |  |  |  |  |  |  |  |  |
| AIC | 2407.1 | 2411.6 | 2407.1 | 2407.1 | 2407.7 | 2410.8 | 2411.7 | 1981.6 |
| BIC | 2533.4 | 2538.0 | 2533.5 | 2533.5 | 2538.5 | 2559.7 | 2560.6 | 2103.0 |
| Log likelihood | -1175.6 | -1177.8 | -1175.6 | -1175.6 | -1174.8 | -1172.4 | -1172.9 | -962.8 |
| Chi-squared | 83.0 | 77.9 | 82.7 | 82.4 | 84.4 | 89.5 | 88.9 | 103.7 |

95% confidence intervals in brackets

^+^ *p* < 0.10, ^*^ *p* < 0.05

Supplement Table 5: Complete results from mixed effects regression mode on Household dietary diversity score (1 month recall)

| Household Dietary Diversity Score | H-Model 1 | H-Model 2 | H-Model 3 | H-Model 4 | H-Model 5 | H- Model 6 (Int. with women’s education) | H- Model 7 (Int. with men’s education) | H- Model 8 (Market; Sub group n =1171) |
| --- | --- | --- | --- | --- | --- | --- | --- | --- |
|  |  |  |  |  |  |  |  |  |
| Women's dietary knowledge | 0.23^*^ |  |  |  | 0.13 | 0.28^*^ | 0.13 | 0.16^+^ |
|  | [0.086,0.38] |  |  |  | [-0.042,0.31] | [0.065,0.49] | [-0.048,0.30] | [-0.021,0.35] |
|  |  |  |  |  |  |  |  |  |
| Women's vitamin knowledge |  | 0.21^*^ |  |  |  |  |  |  |
|  |  | [0.077,0.35] |  |  |  |  |  |  |
|  |  |  |  |  |  |  |  |  |
| Men's dietary knowledge |  |  | 0.24^*^ |  | 0.17^*^ | 0.18^*^ | 0.16 | 0.16^+^ |
|  |  |  | [0.10,0.38] |  | [0.0021,0.34] | [0.013,0.35] | [-0.12,0.45] | [-0.023,0.34] |
|  |  |  |  |  |  |  |  |  |
| Men's vitamin knowledge |  |  |  | 0.23^*^ |  |  |  |  |
|  |  |  |  | [0.068,0.38] |  |  |  |  |
|  |  |  |  |  |  |  |  |  |
| Amhara | 0 | 0 | 0 | 0 | 0 | 0 | 0 | 0 |
|  | [0,0] | [0,0] | [0,0] | [0,0] | [0,0] | [0,0] | [0,0] | [0,0] |
|  |  |  |  |  |  |  |  |  |
| Oromia | -0.32 | -0.31 | -0.33 | -0.33 | -0.34 | -0.35^+^ | -0.32 | -0.46^*^ |
|  | [-0.72,0.082] | [-0.72,0.088] | [-0.74,0.072] | [-0.73,0.075] | [-0.74,0.067] | [-0.75,0.045] | [-0.72,0.081] | [-0.87,-0.041] |
|  |  |  |  |  |  |  |  |  |
| SNNPR | -0.46^*^ | -0.49^*^ | -0.46^*^ | -0.48^*^ | -0.47^*^ | -0.46^*^ | -0.45^*^ | -0.62^*^ |
|  | [-0.90,-0.024] | [-0.93,-0.049] | [-0.90,-0.019] | [-0.93,-0.044] | [-0.91,-0.024] | [-0.89,-0.020] | [-0.90,-0.013] | [-1.08,-0.17] |
|  |  |  |  |  |  |  |  |  |
| Tigray | -1.02^*^ | -1.22^*^ | -1.15^*^ | -1.21^*^ | -1.09^*^ | -1.09^*^ | -1.09^*^ |  |
|  | [-1.48,-0.56] | [-1.68,-0.75] | [-1.61,-0.69] | [-1.67,-0.75] | [-1.55,-0.62] | [-1.55,-0.63] | [-1.55,-0.62] |  |
|  |  |  |  |  |  |  |  |  |
| Wealth quintile 1 | 0 | 0 | 0 | 0 | 0 | 0 | 0 | 0 |
|  | [0,0] | [0,0] | [0,0] | [0,0] | [0,0] | [0,0] | [0,0] | [0,0] |
|  |  |  |  |  |  |  |  |  |
| Wealth quintile 2 | 0.32^*^ | 0.30^*^ | 0.31^*^ | 0.31^*^ | 0.32^*^ | 0.32^*^ | 0.31^*^ | 0.20 |
|  | [0.038,0.61] | [0.019,0.59] | [0.021,0.59] | [0.025,0.60] | [0.032,0.60] | [0.038,0.61] | [0.025,0.59] | [-0.14,0.54] |
|  |  |  |  |  |  |  |  |  |
| Wealth quintile 3 | 0.33^*^ | 0.31^*^ | 0.31^*^ | 0.30^+^ | 0.33^*^ | 0.32^*^ | 0.32^*^ | 0.39^*^ |
|  | [0.026,0.64] | [0.0049,0.62] | [0.0071,0.62] | [-0.0061,0.61] | [0.020,0.63] | [0.015,0.63] | [0.012,0.63] | [0.034,0.74] |
|  |  |  |  |  |  |  |  |  |
| Wealth quintile 4 | 0.27 | 0.25 | 0.26 | 0.26 | 0.27 | 0.26 | 0.27 | 0.25 |
|  | [-0.069,0.60] | [-0.085,0.59] | [-0.075,0.60] | [-0.072,0.60] | [-0.069,0.60] | [-0.078,0.59] | [-0.067,0.60] | [-0.12,0.63] |
|  |  |  |  |  |  |  |  |  |
| Wealth quintile 5 | 0.20 | 0.19 | 0.20 | 0.19 | 0.21 | 0.20 | 0.21 | 0.23 |
|  | [-0.11,0.52] | [-0.12,0.51] | [-0.11,0.52] | [-0.13,0.51] | [-0.11,0.52] | [-0.11,0.52] | [-0.11,0.52] | [-0.13,0.59] |
|  |  |  |  |  |  |  |  |  |
| Women's age | 0.0026 | 0.0024 | 0.0012 | 0.0018 | 0.0016 | 0.00050 | 0.00074 | -0.0035 |
|  | [-0.014,0.019] | [-0.014,0.019] | [-0.015,0.018] | [-0.015,0.018] | [-0.015,0.018] | [-0.016,0.017] | [-0.016,0.017] | [-0.022,0.015] |
|  |  |  |  |  |  |  |  |  |
| Men's age | -0.0059 | -0.0058 | -0.0049 | -0.0054 | -0.0051 | -0.0047 | -0.0049 | -0.0029 |
|  | [-0.018,0.0057] | [-0.017,0.0058] | [-0.017,0.0067] | [-0.017,0.0063] | [-0.017,0.0065] | [-0.016,0.0069] | [-0.017,0.0067] | [-0.016,0.010] |
|  |  |  |  |  |  |  |  |  |
| Total number of HH members | 0.019 | 0.020 | 0.017 | 0.017 | 0.017 | 0.021 | 0.019 | 0.030 |
|  | [-0.028,0.066] | [-0.028,0.067] | [-0.030,0.064] | [-0.030,0.064] | [-0.030,0.065] | [-0.026,0.068] | [-0.028,0.066] | [-0.021,0.081] |
|  |  |  |  |  |  |  |  |  |
| No schooling/missing | 0 | 0 | 0 | 0 | 0 | 0 | 0 | 0 |
|  | [0,0] | [0,0] | [0,0] | [0,0] | [0,0] | [0,0] | [0,0] | [0,0] |
|  |  |  |  |  |  |  |  |  |
| Primary 1 | 0.15 | 0.15 | 0.17 | 0.17 | 0.15 | 0.15 | 0.15 | 0.13 |
|  | [-0.093,0.39] | [-0.089,0.40] | [-0.076,0.41] | [-0.076,0.41] | [-0.087,0.40] | [-0.093,0.39] | [-0.087,0.40] | [-0.13,0.40] |
|  |  |  |  |  |  |  |  |  |
| Primary 2 | 0.23 | 0.23 | 0.25^+^ | 0.25 | 0.24 | 0.24 | 0.25 | 0.28^+^ |
|  | [-0.066,0.53] | [-0.066,0.53] | [-0.041,0.55] | [-0.049,0.54] | [-0.053,0.54] | [-0.058,0.53] | [-0.049,0.54] | [-0.050,0.60] |
|  |  |  |  |  |  |  |  |  |
| Secondary 1, 2, university | 0.036 | 0.032 | 0.060 | 0.079 | 0.033 | -0.0088 | 0.039 | 0.086 |
|  | [-0.45,0.52] | [-0.45,0.51] | [-0.42,0.54] | [-0.40,0.56] | [-0.45,0.52] | [-0.51,0.49] | [-0.44,0.52] | [-0.45,0.62] |
|  |  |  |  |  |  |  |  |  |
| Other | 0.31 | 0.26 | 0.30 | 0.31 | 0.30 | 0.35 | 0.28 | 0.31 |
|  | [-0.21,0.83] | [-0.26,0.78] | [-0.22,0.82] | [-0.21,0.83] | [-0.22,0.82] | [-0.17,0.87] | [-0.24,0.79] | [-0.22,0.84] |
|  |  |  |  |  |  |  |  |  |
| No schooling/missing | 0 | 0 | 0 | 0 | 0 | 0 | 0 | 0 |
|  | [0,0] | [0,0] | [0,0] | [0,0] | [0,0] | [0,0] | [0,0] | [0,0] |
|  |  |  |  |  |  |  |  |  |
| Primary 1 | -0.0023 | 0.0038 | -0.016 | -0.0034 | -0.020 | -0.019 | -0.021 | -0.024 |
|  | [-0.26,0.25] | [-0.25,0.26] | [-0.27,0.24] | [-0.26,0.25] | [-0.28,0.24] | [-0.27,0.24] | [-0.28,0.24] | [-0.31,0.26] |
|  |  |  |  |  |  |  |  |  |
| Primary 2 | 0.40^*^ | 0.39^*^ | 0.35^*^ | 0.37^*^ | 0.36^*^ | 0.35^*^ | 0.33^*^ | 0.46^*^ |
|  | [0.14,0.66] | [0.14,0.65] | [0.092,0.61] | [0.11,0.63] | [0.10,0.62] | [0.093,0.61] | [0.066,0.59] | [0.17,0.75] |
|  |  |  |  |  |  |  |  |  |
| Secondary 1, 2, university | 0.50^*^ | 0.49^*^ | 0.42^*^ | 0.43^*^ | 0.44^*^ | 0.46^*^ | 0.48^*^ | 0.47^*^ |
|  | [0.15,0.86] | [0.14,0.85] | [0.068,0.78] | [0.071,0.79] | [0.086,0.80] | [0.100,0.81] | [0.12,0.85] | [0.084,0.86] |
|  |  |  |  |  |  |  |  |  |
| Other | -0.0085 | 0.0051 | -0.0059 | -0.0031 | -0.015 | -0.013 | -0.058 | -0.19 |
|  | [-0.37,0.36] | [-0.36,0.37] | [-0.37,0.36] | [-0.37,0.36] | [-0.38,0.35] | [-0.38,0.35] | [-0.43,0.31] | [-0.61,0.22] |
|  |  |  |  |  |  |  |  |  |
| No schooling/missing # Women's dietary knowledge |  |  |  |  |  | 0 |  |  |
|  |  |  |  |  |  | [0,0] |  |  |
|  |  |  |  |  |  |  |  |  |
| Primary 1 # Women's dietary knowledge |  |  |  |  |  | -0.21 |  |  |
|  |  |  |  |  |  | [-0.57,0.14] |  |  |
|  |  |  |  |  |  |  |  |  |
| Primary 2 # Women's dietary knowledge |  |  |  |  |  | -0.42^*^ |  |  |
|  |  |  |  |  |  | [-0.84,-0.0099] |  |  |
|  |  |  |  |  |  |  |  |  |
| Secondary 1, 2, university # Women's dietary knowledge |  |  |  |  |  | -0.063 |  |  |
|  |  |  |  |  |  | [-0.72,0.59] |  |  |
|  |  |  |  |  |  |  |  |  |
| Other # Women's dietary knowledge |  |  |  |  |  | -1.27^*^ |  |  |
|  |  |  |  |  |  | [-2.09,-0.45] |  |  |
|  |  |  |  |  |  |  |  |  |
| No schooling/missing # Men's dietary knowledge |  |  |  |  |  |  | 0 |  |
|  |  |  |  |  |  |  | [0,0] |  |
|  |  |  |  |  |  |  |  |  |
| Primary 1 # Men's dietary knowledge |  |  |  |  |  |  | -0.0088 |  |
|  |  |  |  |  |  |  | [-0.40,0.38] |  |
|  |  |  |  |  |  |  |  |  |
| Primary 2 # Men's dietary knowledge |  |  |  |  |  |  | 0.29 |  |
|  |  |  |  |  |  |  | [-0.087,0.67] |  |
|  |  |  |  |  |  |  |  |  |
| Secondary 1, 2, university # Men's dietary knowledge |  |  |  |  |  |  | -0.19 |  |
|  |  |  |  |  |  |  | [-0.63,0.26] |  |
|  |  |  |  |  |  |  |  |  |
| Other # Men's dietary knowledge |  |  |  |  |  |  | -0.47^+^ |  |
|  |  |  |  |  |  |  | [-1.00,0.065] |  |
|  |  |  |  |  |  |  |  |  |
| Distance to the market (minutes) |  |  |  |  |  |  |  | -0.0025^*^ |
|  |  |  |  |  |  |  |  | [-0.0048,-0.00017] |
| *AIC* | 5428.8 | 5429.0 | 5427.1 | 5430.6 | 5426.9 | 5422.6 | 5425.0 | 4569.9 |
| *BIC* | 5544.1 | 5544.3 | 5542.4 | 5545.9 | 5547.4 | 5564.1 | 5566.5 | 4686.4 |
| Log likelihood | -2692.4 | -2692.5 | -2691.6 | -2693.3 | -2690.5 | -2684.3 | -2685.5 | -2262.0 |
| Chi-squared | 79.2 | 78.5 | 80.4 | 77.2 | 82.7 | 96.3 | 93.3 | 66.9 |

95% confidence intervals in brackets

^+^ *p* < 0.10, ^*^ *p* < 0.05
